# N-terminal fusion length: The key to reliable and context-preserving regulatory sequence characterization

**DOI:** 10.1016/j.synbio.2026.05.020

**Published:** 2026-07-22

**Authors:** Sen Yang, Ye Liu, Anqi Li, Shenhui Wang, Jiawen Cui, Huanhuan Liu, Meng Wang, Yufeng Mao

**Affiliations:** a Tianjin University of Science and Technology, Tianjin, 300457, China; b College of Food Science and Engineering, Tianjin University of Science and Technology, Tianjin, 300457, China; c College of Biotechnology, Tianjin University of Science and Technology, Tianjin, 300457, China; d Tianjin Institute of Industrial Biotechnology, Chinese Academy of Sciences, Tianjin, 300308, China; e State Key Laboratory of Engineering Biology for Low-Carbon Manufacturing, Tianjin, 300308, China

**Keywords:** Regulatory sequences, N-terminal fusion, Gene expression, *E. coli*, Synthetic biology

## Abstract

Regulatory sequences are commonly characterized using fluorescent reporters, yet how N-terminal coding context shapes these measurements has not been systematically quantified. Here, we evaluated the impact of N-terminal fusion length (45-180 bp) from four genes (*lacZ*, *icd*, *zwf*, *bfp*) on GFP reporter expression driven by 15 different promoter-RBS combinations in *E. coli* with normalized fluorescence, enzymatic activity assays and transcription analysis for a representative subset of constructs. Our results demonstrate that N-terminal fusion critically determines the reliability of regulatory-sequence characterization in target-gene-specific coding contexts, with strong gene- and length-dependent effects. Fusions as short as 45 bp failed to rescue context-sensitive cases. However, among the tested fusion lengths, fusions of 90 bp or longer achieved strong correlations (mean R^2^ > 0.75) between reporter fluorescence and target protein activity. Among the factors examined, N-terminal mRNA secondary structure showed a closer association with these fusion-length-dependent effects than transcription or translation initiation changes. This practical, context-preserving fusion strategy provides cost-effective guidance for scalable and accurate regulatory sequence profiling.

## 1 Introduction

Precise regulation of gene expression depends on well-characterized regulatory sequences and is important for synthetic biology [[1], [2], [3]]. Promoters and ribosome-binding sites (RBSs) are classical and essential regulatory elements that control gene expression by regulating the initiation of transcription and translation, respectively [[4], [5], [6], [7]]. Numerous promoter and RBS libraries have been constructed and characterized to enable fine-tuning of gene expression across broad strength ranges [8]. The characterization of regulatory sequences typically relies on a limited number of easily detectable reporter proteins, such as fluorescent proteins [7,9,10] and β-galactosidase [11,12]. This practice implicitly assumes that regulatory sequences exhibit similar behaviors across different target genes. However, increasing evidence indicates that gene expression is highly context-dependent, and shaped by a complex interplay of multiple factors during transcription and translation [4,[13], [14], [15], [16], [17]]. As a result, regulatory sequences optimized or characterized for one gene frequently perform unpredictably or sub-optimally when reused for other genes [[18], [19], [20], [21]], posing a major obstacle to reliable multi-gene expression and pathway optimization. Therefore, accurate characterization of regulatory sequences requires an expression context that closely matches the intended target gene.

The coding sequences of different target genes, particularly the N-terminal coding sequence, are increasingly recognized as a critical determinant of gene expression [13,16,[22], [23], [24], [25], [26], [27], [28], [29]]. Importantly, these coding contexts do not merely act as passive recipients of upstream regulatory sequences, but actively interact with them to shape transcriptional and translational outcomes. As a result, identical regulatory sequences can drive markedly different expression levels when coupled to different target genes. Mechanistically, the N-terminal coding sequence has been linked to transcript stability [13,22] and translation initiation [6,30,31] through factors such as codon usage and mRNA secondary structure. Yet, the dominant sequence window remains debated. Prior studies have proposed distinct sequence windows including the −4 to +37 region relative to the start codon [29], the −30 to +90 region [10], the region around nucleotide +10 [28] and the first 18 nucleotides [13]. This lack of consensus complicates the rational design of context-preserving reporter systems for reliable regulatory sequence characterization.

To mitigate the context dependence between regulatory sequences and target genes, a commonly adopted strategy is to fuse the N-terminal coding sequence of the target gene to a reporter protein via a flexible linker [18,25,32,33]. In practice, the first ∼180 bp of the coding sequence is frequently used, largely based on empirical convention rather than a defined mechanistic rationale. In our previous study, this strategy was shown to improve regulatory characterization, as the fluorescence intensities of GFP fusion proteins carrying the first 180 bp of metabolic gene (*icd* and *zwf*) closely correlated with their corresponding enzymatic activities across different regulatory sequences [18]. Despite its effectiveness, the use of a 180 bp N-terminal fusion introduces practical limitations. From an engineering perspective, the extended fusion length substantially increases synthesis cost and constrains library design. In large-scale applications such as massively parallel reporter assays (MPRAs) [34,35] and fluorescence-activated cell sorting coupled with sequencing (FACS-seq) [36,37], the finite length of synthetic oligonucleotide pools severely limits the number and diversity of regulatory sequences that can be interrogated. More fundamentally, given that only the proximal region of the coding sequence is believed to dominantly influence gene expression, it remains unclear whether such a long fusion is necessary to faithfully capture regulatory compatibility. The minimal N-terminal sequence length required to preserve accurate regulatory characterization has not been systematically examined.

Here, we systematically investigate how N-terminal coding sequence length influences the accurate characterization of the strength of regulatory sequences, which in this study refer specifically to promoter-RBS combinations. We constructed a comprehensive library that combines multiple regulatory sequences with N-terminal fusions of varying lengths (45, 90, 135, and 180 bp) derived from four target genes (*lacZ*, *icd*, *zwf* and *bfp*). Using an automated, high-throughput workflow, we quantified reporter fluorescence across growth stages and benchmarked reporter outputs against target protein function and transcript abundance through parallel measurements of fluorescence, enzymatic activity, and mRNA levels for a representative subset of constructs. Our results demonstrate that N-terminal fusion length critically affects the reliability of regulatory-sequence characterization in target-gene-specific coding contexts, with truncated fusions of approximately 90 bp or longer achieving performance comparable to the conventional 180 bp strategy within the tested design space. Moreover, systematic analysis across regulatory and coding sequence combinations reveals gene- and length-dependent effects that cannot be captured using unfused reporter systems, highlighting the complex interplay between regulatory sequences and N-terminal coding context. Together, this work establishes a scalable and mechanistically informed framework for regulatory sequence characterization and provides practical guidance for high-throughput expression library construction and evaluation.

## 2 Materials and methods

### 2.1 Bacterial strains and culture conditions

Strains used in this study are listed in Supplementary Table 1. *E. coli* DH5α was used for molecular cloning and manipulation. *E. coli* DB3.1 was used for construction of the plasmids carrying *ccdB* cassette. *E. coli* MG1655 was used as host for fluorescence intensity measurement and enzyme activity assays. *E. coli* strains were cultivated at 37 °C in Luria-Bertani (LB) medium or M9 minimal medium (6.8 g/L Na_2_PO_4_, 3 g/L KH_2_PO_4_, 0.5 g/L NaCl, 1 g/L NH_4_Cl, 2 mM MgSO_4_, 100 μM CaCl_2_, with 10 g/L glucose). Ampicillin (Amp, 100 mg/L) was added for *E. coli* strains carrying the pSC101-derived plasmids.

### 2.2 Plasmid construction

Plasmids used in this study are listed in Supplementary Table 1. The pSC101-ccdB-GFP [18], a previously reported backbone plasmid with a pSC101 replicon and a GFP expression cassette, was further optimized in this study. To enable more accurate characterization of regulatory sequences, a constitutively expressed mCherry expression cassette driven by promoter pL_M1-37 [38] was incorporated as an internal reference. In addition, two bidirectional transcriptional terminators were placed upstream and downstream of the GFP expression cassette to eliminate potential interference from local transcription events occurring outside the cassette. The resulting plasmid was named pSC101-ccdB-GFP-mCherry-opt.

To construct the GFP fusion expression plasmid library, synthetic DNA fragments containing distinct regulatory sequences and N-terminal coding sequences of varying lengths from various target genes (*lacZ*, *icd*, *zwf*, *bfp*, *gdhA*, *icd-P.p*, *b2943*, *b3952*) were cloned into the pSC101-ccdB-GFP-mCherry-opt backbone by replacing the *ccdB* cassette. The 15 regulatory sequences used in this study were designated RS1-RS15. Detailed information for these regulatory and N-terminal coding sequences is provided in Supplementary Data, including construct IDs, promoter IDs, RBS IDs, regulatory-sequence IDs and their corresponding sequences. Synthetic genes were ordered through the GenScript Gene Synthesis service (GenScript, China).

To facilitate the construction of expression plasmids containing different regulatory sequences and target genes, a set of pSC101-RSX-*ccdB*-mCherry-opt backbone plasmids (where RSX denotes RS1-RS15) was first constructed by introducing 15 distinct regulatory sequences upstream of the *ccdB* gene and removing the *gfp* gene in pSC101-ccdB-GFP-mCherry-opt. GFP expression plasmids lacking N-terminal fusions were subsequently constructed by replacing the *ccdB* gene with *gfp* in pSC101-RSX-*ccdB*-mCherry-opt. In addition, this set of pSC101-RSX-*ccdB*-mCherry-opt backbone plasmids was used to construct plasmids expressing β-galactosidase (β-GAL), isocitrate dehydrogenase (ICDH), glucose-6-phosphate dehydrogenase (G6PDH), blue fluorescent protein (BFP), glutamate dehydrogenase (GDH), and ICDH from *Pseudomonas putida* KT2440 by replacing *ccdB* with *lacZ*, *icd*, *zwf*, *bfp*, *gdhA*, and *icd-P.p*, respectively.

For the synonymous mutational experiments, in-house Python scripts were used to generate approximately 300,000 synonymous variants for each of the four target genes (*lacZ*, *icd*, *zwf* and *bfp*) under the control of RS8, with altered mRNA secondary structures while preserving the encoded amino acid sequences. From these variants, one representative sequence for each gene exhibiting an obvious change in predicted minimum free energy (MFE) relative to the original sequence was selected (Supplementary Table 2). DNA fragments containing the RS8 sequence and N-terminal coding sequences of varying lengths from these selected mutant genes were then synthesized and cloned into the pSC101-ccdB-GFP-mCherry-opt backbone by replacing the *ccdB* cassette (Supplementary Data).

Additionally, the pSC101-mCherry-opt plasmid was constructed by removing both the *ccdB* gene and the GFP expression cassette to serve as a negative control.

All primers used for plasmids construction are listed in Supplementary Table 3. DNA fragments were amplified using Q5 High-Fidelity DNA Polymerase (NEB, USA), purified with the Universal DNA Purification Kit (TIANGEN, China), and assembled using the ClonExpress II One Step Cloning Kit (Vazyme, China).

### 2.3 Comparative analysis of fluorescence normalization methods

To compare the performance of different fluorescence normalization methods, three GFP expression plasmids (without N-terminal fusions) driven by regulatory sequences with distinct strengths (RS4, RS6 and RS12) were selected and transformed in *E. coli* MG1655. Single colonies were picked and cultured in M9 minimal medium. The cultured cells in the stationary phase were harvested and serially diluted to generate a range of cell densities. The fluorescence intensities of GFP (excitation: 460 nm, emission: 510 nm) and mCherry (excitation: 580 nm, emission: 615 nm), as well as the OD_600nm_ of the diluted cells, were measured using the BioTek Neo2 microplate reader (BioTek, USA).

For evaluation of the expression stability of mCherry, GFP expression plasmids (without N-terminal fusions) with 15 distinct regulatory sequences were selected and transformed in *E. coli* MG1655. Single colonies were picked and cultured overnight in LB medium, then the cultured cells were inoculated into M9 minimal medium with an initial OD_600nm_ of 0.2. After 6 h of growth, GFP and mCherry fluorescence as well as cell density (OD_600nm_) were measured.

### 2.4 Automated high-throughput fluorescence characterization of GFP fusion proteins

To enable scalable assessment, we implemented an automated, high-throughput workflow described previously employing our integrated robotic system [18]. This workflow encompassed the plasmid transformation, cell culture, and multi-timepoint measurements. Competent *E. coli* MG1655 cells were prepared using a commercial transformation kit (TaKaRa, Japan). Plasmid transformation was then performed in 96-well PCR plates according to the published protocol. Briefly, plasmids were mixed with competent cells, incubated on ice for 10 min, subjected to a 42 °C heat shock for 90 s, and then transferred to SOC recovery medium in 96-deep-well plates for a 1 h of incubation at 37 °C. Subsequently, cells were centrifuged, resuspended, and plated onto LB agar plate using the QPix 420 system (Molecular Devices, USA).

Single colonies were automatically picked into 96-deep-well plates containing LB medium (1000 μL per well) and cultured overnight at 37 °C with shaking at 800 rpm (INFORS HT, Switzerland). Three biological replicates were prepared for each construct. Overnight cultures were then inoculated into new 96-deep-well plates containing M9 minimal medium (1000 μL per well) with an initial OD_600nm_ of 0.2, followed by incubation at 37 °C with shaking at 800 rpm. GFP and mCherry fluorescence were measured after 6 h, 12 h and 24 h of growth, with excitation/emission wavelengths of 460/510 nm for GFP and 580/615 nm for mCherry. For each measurement, 100 μL of cell culture was transferred to 96-well microplates using a liquid-handling workstation, and fluorescence was measured using a SpectraMax iD3 microplate reader (Molecular Devices, USA). Wells containing M9 minimal medium alone were included in each plate for background subtraction.

For data processing, fluorescence values were background-corrected by subtracting the signal of culture medium. Normalized GFP/mCherry values were then calculated, and the residual background measured from the negative control strain harboring pSC101-mCherry-opt was subtracted to obtain final normalized reporter outputs.

### 2.5 Enzyme activity assays

*E. coli* MG1655 strains harboring different expression plasmids were cultured overnight in LB medium, then inoculated into M9 minimal medium with an initial OD_600nm_ of 0.2. Cells were harvested after 6, 12, and 24 h of growth for enzyme activity assays, except for BFP. For BFP, the fluorescence intensities (excitation: 399 nm, emission: 456 nm) were measured and normalized to mCherry values, as described above for GFP. *E. coli* MG1655 harboring the pSC101-mCherry-opt plasmid was used as the negative control. Three biological replicates were prepared for each construct.

Cell pellets were resuspended in ice-cold Tris-HCl buffer (20 mM Tris, 150 mM NaCl, pH 7.6). Cell lysis was performed at 4 °C by adding TieChui *E. coli* Lysis Buffer (ACE Biotechnology, China) to the resuspension at a 1:9 (v/v) ratio. Lysates were centrifuged at 4 °C, and supernatants (crude extract) were collected for subsequent assays. Total protein concentration of each crude extract was determined using a BCA Protein Assay Kit (QuaYad, China).

The activities of β-GAL, ICDH, G6PDH, GDH were determined using commercial assay kits (Solarbio, China) according to the manufacturer's instructions. Automated liquid handling was performed using a Beckman i7 96-channel pipettor (Beckman-Biomek, USA). Reaction progress was monitored using a SpectraMax iD3 microplate reader (Molecular Devices). β-GAL activity was quantified by measuring the rate of p-nitrophenol formation at 400 nm, whereas ICDH and G6PDH activities were determined by monitoring NADPH formation at 340 nm, and GDH activity was determined by monitoring NADH formation at 340 nm. One unit of β-GAL activity was defined as the amount of enzyme that produces 1 nmol of p-nitrophenol per hour. One unit of ICDH or G6PDH activity was defined as the amount of enzyme that generates 1 nmol of NADPH per minute. One unit of GDH activity was defined as the amount of enzyme that generates 1 nmol of NADH per minute. Activities obtained for each construct were background-corrected by subtracting the corresponding negative-control value from strain harboring the pSC101-mCherry-opt plasmid.

### 2.6 RT-qPCR analysis

For transcript quantification, *E. coli* MG1655 strains harboring GFP fusion expression plasmids were cultured overnight in LB medium, then inoculated into M9 minimal medium with an initial OD_600nm_ of 0.2. Cells were harvested after 6 h of growth. Approximately 1 × 10^9^ cells from each sample were used for total RNA extraction. Total RNA was extracted from cultured cells using an automated magnetic bead-based system. Specifically, cells were pre-treated with the TGuide Smart Universal RNA Kit (TIANGEN, China) and then processed on a TGuide S32 Automated Nucleic Acid Extraction System (TIANGEN, China). cDNA was synthesized from total RNA using the ReverTra Ace qPCR RT Master Mix (Toyobo, Japan) with the following reverse-transcription program: 37 °C for 15 min, 50 °C for 5 min, and 98 °C for 5 min qPCR was performed using the SYBR Green Realtime PCR Master Mix (Toyobo, Japan) on a LightCycler 480 Real-Time PCR System (Roche, Germany). The qPCR cycling program was as follows: 95 °C for 5 min, followed by 45 cycles of 95 °C for 10 s, 60 °C for 10 s, and 72 °C for 10 s. Melting-curve analysis was then performed using 95 °C for 5 s, 65 °C for 1 min, and 40 °C for 30 s. All primers used are listed in Supplementary Table 3. Relative mRNA levels of the target genes were calculated using the comparative ΔΔCt method [39]^.^

## 3 Results

### 3.1 Optimizing fluorescence normalization for regulatory sequence characterization

For high-throughput characterization of regulatory sequences, fluorescence intensities are commonly normalized to cell density (e.g., fluorescence/OD_600nm_) to compensate growth variation. However, this method is sensitive to measurement noise, particularly at very low or high cell densities (Supplementary Fig. 1), and fails to correct for cell-to-cell heterogeneity arising from plasmid copy number variation.

**Fig. 1:**
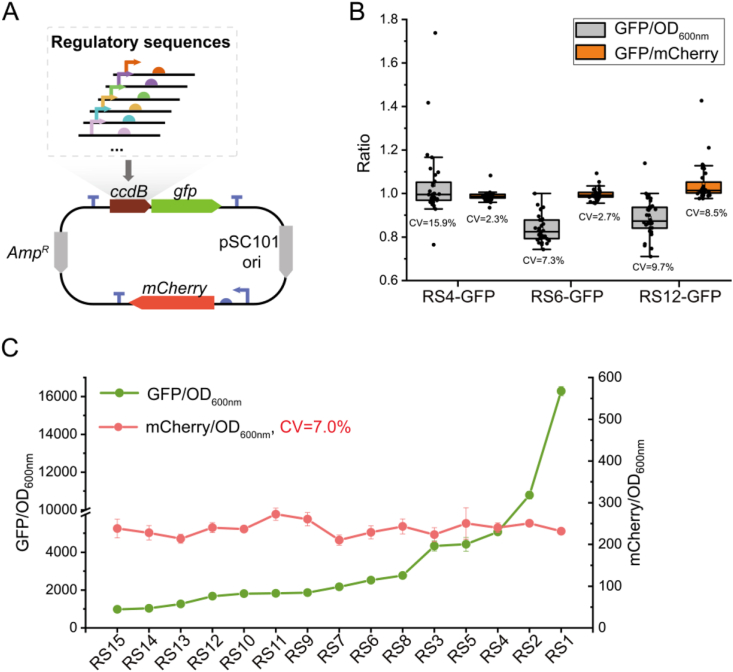
Optimization of fluorescence normalization for regulatory sequence characterization. (A) Schematic of the optimized reporter plasmid containing a constitutively expressed mCherry cassette used as an internal normalization control. (B) Comparison of coefficients of variation for different fluorescence normalization methods. Cells exhibiting three distinct GFP expression levels were serially diluted to obtain a range of cell densities. The ratio was calculated for each dilution by dividing its GFP/OD_600nm_ or GFP/mCherry value by that of the undiluted culture. The box represents the data point between first quartile and third quartile, and the central line indicates the median. (C) Response of mCherry expression to changes in GFP expression.

To improve measurement robustness, we constructed an optimized low-copy plasmid, pSC101-ccdB-GFP-mCherry-opt, in which a constitutively expressed mCherry cassette driven by promoter pL_M1-37 serves as an internal reference (Fig. 1A). We benchmarked normalization performance by serially diluting cultures spanning three representative GFP expression levels and quantifying how normalization values varied across the resulting density range. Compared with GFP/OD_600nm_, GFP/mCherry normalization consistently exhibited lower coefficients of variation (CV) across dilutions (Fig. 1B, Supplementary Fig. 1), indicating improved resistance to density-dependent measurement artifacts.

Considering that the expression of different proteins competes for shared intracellular transcriptional and translational resources [40], we further examined whether the mCherry reference remained stable across a wide range of GFP outputs. GFP expression was driven by 15 previously defined regulatory sequences, designated RS1 to RS15 in descending order of strength [18]. The results showed that mCherry expression remained highly stable across constructs spanning a ∼17-fold range of GFP expression, with a CV of only 7% (Fig. 1C). These results establish GFP/mCherry normalization as a robust and reliable strategy for quantitative characterization of regulatory sequences.

### 3.2 N-terminal fusion length determines the accuracy of regulatory sequence characterization

**Fig. 2:**
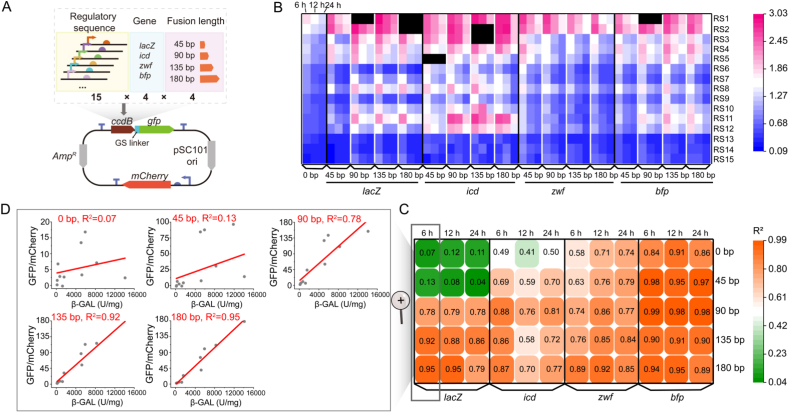
N-terminal fusion length influences regulatory sequence characterization. (A) Design of GFP reporter library containing N-terminal coding sequences of varying lengths from four target genes, each driven by 15 pre-characterized regulatory sequences. (B) Heat map showing log_10_-transformed fluorescence levels (log_10_(GFP/mCherry+2)) of different GFP fusion constructs. (C) Correlation (R^2^) analysis between normalized GFP/mCherry fluorescence and the functional outputs of the corresponding target proteins across fusion constructs. (D) Detailed correlation analysis between normalized GFP/mCherry fluorescence and β-GAL enzyme activity for constructs with varying N-terminal fusion lengths at 6 h of growth.

To systematically explore how N-terminal fusion length influences regulatory sequence characterization, four target genes with diverse functions were selected: *lacZ*, *icd*, *zwf* and *bfp* (Fig. 2A), encoding β-galactosidase (β-GAL), isocitrate dehydrogenase (ICDH), glucose-6-phosphate dehydrogenase (G6PDH) and blue fluorescent protein (BFP), respectively. For each gene, N-terminal coding sequences of defined lengths (45, 90, 135 and 180 bp) were fused upstream of GFP via a flexible linker (GGGGS). The flexible linker was introduced to reduce steric hindrance between the N-terminal peptide and GFP, thereby minimizing potential perturbations to GFP folding and fluorescence maturation. These GFP fusion constructs were used for reporter-based fluorescence characterization, whereas the functional outputs of the corresponding target proteins were evaluated independently using full-length β-GAL, ICDH, G6PDH, or BFP expression constructs. Each fusion construct was expressed under the control of the same set of 15 regulatory sequences, generating a comprehensive library of context-varied GFP fusion reporters. In total, 233 plasmids were successfully constructed, while 7 constructs, predominantly driven by the strong regulatory sequences RS1 and RS2, could not be obtained (Supplementary Data). This is likely due to toxicity associated with high-level expression of the GFP fusion proteins [41,42].

GFP and mCherry fluorescence signals were measured at three growth stages: exponential phase (6 h), early stationary phase (12 h) and middle stationary phase (24 h) (Supplementary Fig. 2). Across the library, mCherry remained stable (Supplementary Fig. 3), whereas the GFP signals exhibited pronounced, fusion-dependent shifts in expression profiles across regulatory sequences (Fig. 2B). This indicates that the observed differences primarily arise from fusion-dependent effects rather than global changes in cellular expression resources.

To assess whether reporter fluorescence faithfully reflects target gene expression, we compared the normalized GFP/mCherry outputs with functional outputs of the corresponding target proteins. While unfused GFP fluorescence correlated well with BFP fluorescence (R^2^ = 0.84–0.91), it showed no or relatively lower correlation with the enzymatic activities of β-GAL (R^2^ = 0.07–0.12), ICDH (R^2^ = 0.41–0.50) or G6PDH (R^2^ = 0.58–0.74) (Fig. 2C). Notably, fusion of short N-terminal sequences (45 bp) failed to substantially improve these correlations, particularly for the context-sensitive *lacZ* constructs (Fig. 2D). In contrast, extending the N-terminal fusion length to 90 bp consistently restored strong correspondence between reporter fluorescence and target protein activity across genes and growth stages, with R^2^ values of at least 0.74 in all cases (Fig. 2C). Further extension to 135 bp or 180 bp provided only limited additional improvement in most cases. Thus, within the tested design space, the 90 bp fusion represented a practical effective window among the tested fusion lengths that provided a favorable balance between reporter-target correlation and construct length.

### 3.3 N-terminal fusions reshape the apparent strength landscape of regulatory sequences

**Fig. 3:**
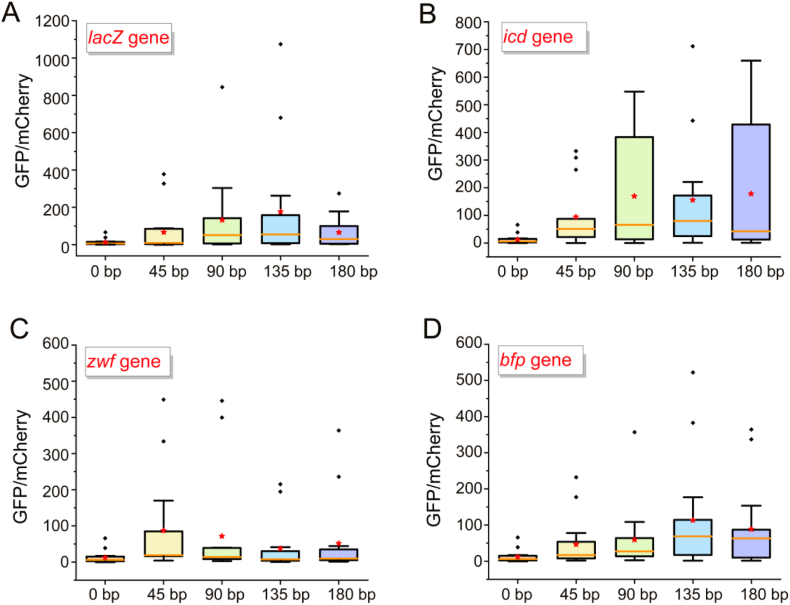
Distribution of normalized expression strengths for all 15 regulatory sequences across increasing N-terminal fusion lengths for the four target genes at 6 h of growth (A-D, *lacZ*, *icd*, *zwf* and *bfp*, respectively). Each panel corresponds to one target gene. The box represents the data point between first quartile and third quartile, the central orange line indicates the median, and the red star represents the mean.

Beyond improving correspondence between reporter output and target gene expression, N-terminal fusion markedly reshaped the apparent strength landscape of regulatory sequences at protein level. When unfused GFP reporters were used, the effective dynamic range of the 15 regulatory sequences was compressed, masking differences that became evident upon fusion with target gene N-terminal sequences (Figs. 2B and 3).

To quantitatively describe this redistribution, we compared the spread of normalized fluorescence values across regulatory sequences for each fusion length and gene (Supplementary Fig. 4). Across all four genes, N-terminal fusion consistently expanded the observable strength change range relative to unfused GFP constructs, although the magnitude of this effect was strongly gene-dependent (Fig. 3A–D). Among the tested genes, *lacZ*-derived fusions exhibited the most pronounced redistribution of regulatory strengths (4.1–16.2 fold compared with unfused GFP reporters, Supplementary Fig. 4), followed by *icd* (5.0–10.7 fold), *zwf* (3.2–6.7 fold) and *bfp* (3.5–7.9 fold). These differences indicate that intrinsic properties encoded within the N-terminal coding context determine how regulatory sequences are functionally manifested.

**Fig. 4:**
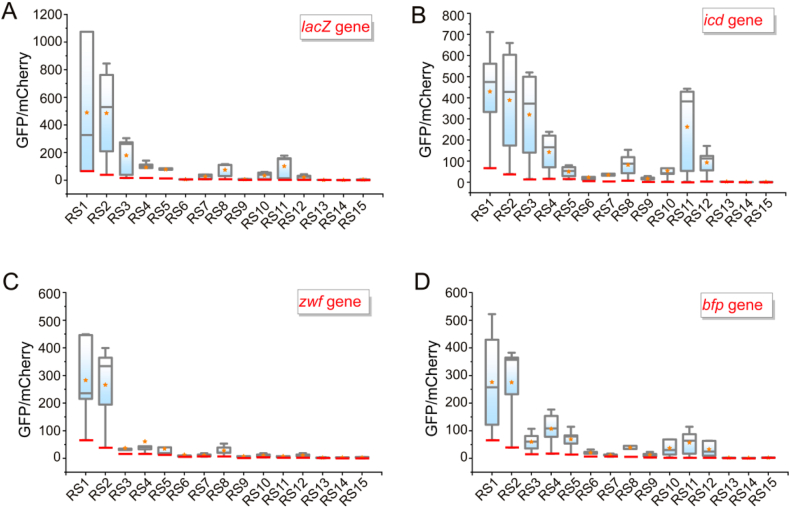
Regulatory-sequence-specific expression changes upon N-terminal fusion. Changes in normalized expression strength for individual regulatory sequences following fusion with N-terminal coding sequences of varying lengths from the four target genes at 6 h of growth (A-D, *lacZ*, *icd*, *zwf* and *bfp*, respectively). Each panel shows the fusion-length-dependent behavior of all regulatory sequences for one target gene. The box represents the data point between first quartile and third quartile, the central gray line indicates the median, the orange star represents the mean, and the red line denotes the fluorescence of unfused GFP.

Regulatory sequences of different strengths responded heterogeneously to N-terminal fusion. Strong regulatory sequences such as RS1 and RS2 exhibited large variability across fusion contexts (Fig. 4 and Supplementary Fig. 5). In contrast, weak regulatory sequences (e.g., RS13, RS14 and RS15) showed relatively stable expression regardless of fusion length or gene identity (Fig. 4 and Supplementary Fig. 5). Intermediate-strength regulatory sequences displayed gene-specific behavior. For example, RS3 and RS11 showed a significantly broad range of variation when fused to *icd* N-terminal sequences, whereas the same regulatory sequences showed much smaller changes when fused to *zwf*. These patterns support the view that strong regulatory sequences expose and magnify coding-context constraints, whereas under weak regulatory input, initiation becomes rate-limiting, reducing the impact of downstream coding features on protein output.

To examine whether the redistribution of apparent regulatory strength is also reflected at the transcriptional level, we analyzed mRNA abundances for representative regulatory sequences displaying distinct fluorescence behaviors, including RS2 (strong strength with broad dynamic range), RS3 (intermediate strength with broad dynamic range) and RS5 (intermediate strength with narrow dynamic range) (Supplementary Fig. 5). For each regulatory sequence, constructs combining four target genes and different N-terminal fusion lengths were quantified by RT-qPCR during exponential growth. Consistent with the protein-level measurements, regulatory sequences exhibiting broader dynamic ranges in fluorescence also displayed wider ranges of transcript abundance, with RS2 showing the highest mRNA levels and the greatest variability and RS5 exhibiting the lowest levels and the narrowest range (Supplementary Fig. 6A). However, transcriptional differences were substantially more compressed than those observed at the protein level, and the correlation between mRNA abundance and fluorescence intensity varied across regulators, ranging from weak to moderate (r = 0.29–0.53, Supplementary Fig. 6B–D). These results indicate that while N-terminal fusion can modulate regulatory sequence output at the transcriptional level, transcription alone is insufficient to account for the extensive reshaping of regulatory strength observed at the protein level, pointing to dominant contributions from post-transcriptional mechanisms.

Together, these results demonstrate that N-terminal fusion reshapes the apparent strength landscape of regulatory sequences primarily at the protein level. While transcriptional output partially mirrors this redistribution, it cannot account for its magnitude, suggesting that post-transcriptional mechanisms play a dominant role. This context-dependent behavior provides a mechanistic basis for why regulatory sequences characterized using unfused reporters often fail to generalize across target genes, particularly for strong ones.

### 3.4 Molecular determinants underlying fusion-length-dependent expression effects

To investigate the molecular basis of the fusion-length-dependent effects observed above, we systematically examined multiple candidate factors at both transcriptional and translational levels. Specifically, we assessed whether transcription initiation, translation initiation and mRNA secondary structure could account for the altered expression patterns associated with different N-terminal fusion lengths.

#### 3.4.1 Transcription initiation does not account for fusion-length-dependent effects

We first evaluated whether fusion of different N-terminal coding sequences altered transcription initiation. Using the Promoter Calculator [4], an automated model predicting site-specific transcription initiation rates, we found that both the dominant TSS position and the predicted transcription initiation rate remained unchanged under a fixed regulatory sequence (e.g., RS2, RS3 or RS5) with different N-terminal fusions (Supplementary Fig. 7). Because internal TSSs within coding sequences can modulate transcript abundance or generate interfering antisense RNAs [43,44], we further predicted internal TSSs within the first 180 bp of the coding sequences of four genes (*icd*, *lacZ*, *zwf* and *bfp*). No additional TSSs with substantial transcription initiation activity (>∼3000 au reported in several literature [4]) were predicted in *icd*, *lacZ* or *zwf* (Supplementary Fig. 7) which was consistent with previous *E. coli* transcriptome study [45].

In contrast, when the same regulatory sequences were combined with *bfp*, a prominent additional TSS was consistently predicted on the antisense strand in the region adjacent to the regulatory sequence-*bfp* junction, with a relatively stable predicted initiation rate of ∼6000 a.u. across regulatory sequences. This putative junction-associated antisense TSS could generate antisense transcripts near the 5′ end of the fusion, potentially influencing sense-transcript stability and/or local RNA architecture. However, this could not account for the relatively context-insensitive behavior of *bfp*-derived constructs observed in our reporter library (Supplementary Fig. 5A). Nonetheless, junction-generated TSSs may introduce an additional regulatory layer in specific context combinations. These results suggest that fusion-dependent expression differences are unlikely to primarily arise from changes in transcription initiation.

#### 3.4.2 Translation initiation efficiency contributes to baseline expression differences but does not explain length dependence

**Fig. 5:**
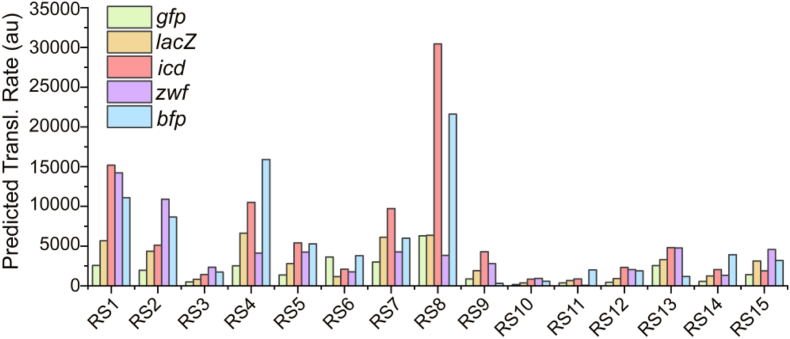
Translation initiation rates were predicted for each GFP fusion construct using the RBS Calculator v2.1. For a given target gene, the predicted translation initiation rate remained essentially unchanged across different N-terminal fusion lengths.

We next examined whether fusion-length-dependent effects could be attributed to altered translation initiation efficiency. Translation initiation rates for all fusion constructs were predicted using the Ribosome Binding Site (RBS) Calculator [6], which can estimate the translation initiation rate for each potential start codon within an mRNA transcript. Across most regulatory sequences, the predicted translation initiation rate of unfused GFP was lower than that of the four target genes (with the exception of RS6) (Fig. 5), potentially accounting for the increased fluorescence observed upon N-terminal fusion (Fig. 3 and Supplementary Fig. 5B). However, the predicted translation initiation rates for a given target gene did not vary with fusion length, which cannot explain why constructs driven by strong regulatory sequences remained highly fusion-length-sensitive. Moreover, predicted initiation rates showed only weak to moderate correlations with the measured fluorescence intensities across regulatory sequences (with a maximum r = 0.37, observed for the 45 bp fusion in Supplementary Fig. 8).

We also examined the potential contribution of alternative in-frame downstream start codons introduced by N-terminal fusions, which may enhance translation of the downstream GFP protein [16]. However, such additional translation initiation events were observed only in *bfp* (Supplementary Fig. 9) and its occurrence did not correspond to systematic increases in expression (Supplementary Fig. 5A), nor did they account for gene- or regulatory-sequence-specific fusion-length effects (Figs. 3D and 4D). These observations indicate that while translation initiation efficiency influences baseline expression levels, it cannot explain the systematic and fusion-length-dependent reshaping of regulatory sequence output.

#### 3.4.3 mRNA secondary structure substantially contributes to fusion-length-dependent expression variability

**Fig. 6:**
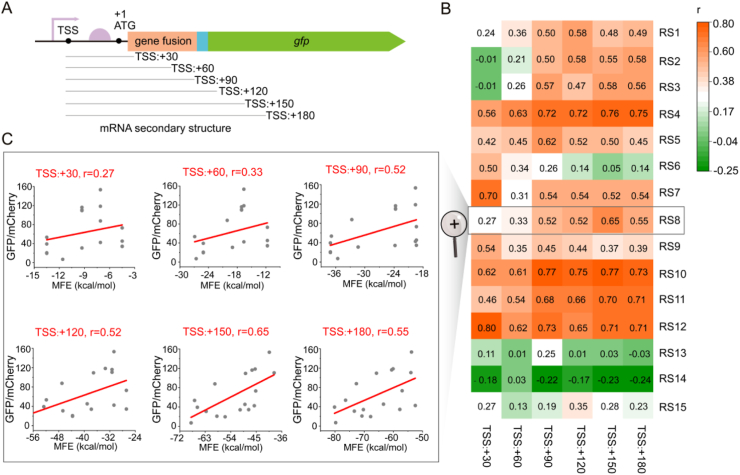
Effects of mRNA secondary structure on N-terminal fusion-dependent expression changes. (A) The selected mRNA sequence region used for calculating the minimum free energy (MFE) of mRNA folding. (B) Correlation between normalized GFP/mCherry fluorescence (at 6 h of growth) and predicted MFE across GFP fusion constructs (containing N-terminal coding sequences of varying lengths from the four target genes), with analysis performed separately for each regulatory sequence group. (C) Detailed correlation analysis across different predicted regions of all GFP fusion constructs under the control of RS8. Correlation coefficients were categorized as negligible (0 ≤ |r| < 0.1), weak (0.1 ≤ |r| < 0.3), moderate (0.3≤ |r| <0.5), strong (0.5 ≤ |r| < 0.8), or very strong (0.8 ≤ |r| ≤ 1), according to commonly used thresholds for interpreting linear correlations.

Finally, we examined whether fusion-length-dependent expression effects could be explained by changes in mRNA secondary structure. Given the well-established influence of RNA folding near the start codon on ribosome accessibility and translation efficiency [10,28,29], minimum free energy (MFE) values were calculated using RNAfold [46] for progressively extended regions spanning from the transcription start site (TSS) to +30, +60, +90, +120, +150 and + 180 nucleotides (Fig. 6A).

When MFE was calculated for the short window from the TSS to +30 nucleotides, more than half of the regulatory sequences exhibited moderate to strong positive correlations (0.3-0.8) between normalized fluorescence intensity and MFE (Fig. 6B). This indicates that local RNA structures proximal to the start codon can influence translation efficiency. However, these correlations were highly regulator-dependent and insufficient to explain the systematic fusion-length-dependent expression patterns observed across constructs.

As the MFE calculation window was extended, correlations between fluorescence intensity and MFE generally strengthened and reached maximal values when the analyzed region spanned approximately +90 to +150 nucleotides, after which correlations plateaued or showed no further improvement for most regulatory sequences, with the exceptions of RS6 and RS7. For example, under the control of RS8, the correlation coefficient increased from 0.27 for the +30 nucleotides window to 0.65 when the window was extended to +150 nucleotides, where the maximal correlation was observed (Fig. 6C). When regions extending beyond +90 nucleotides were analyzed, strong positive correlations were observed for 10 out of 15 regulatory sequences, accompanied by two additional moderate correlations (RS9 and RS15).

In contrast, weak regulatory sequences such as RS13, RS14 and RS15 consistently exhibited only negligible or weak correlations across all window lengths. This behavior suggests that under weak regulatory drive, transcription or translation initiation might become the dominant limiting factor, thereby constraining the extent to which mRNA secondary structure can modulate expression. RS6 displayed a distinct pattern: although a strong positive correlation was observed when only the +30 nucleotides region was considered, the correlation weakened as downstream regions were included. This suggests that expression under RS6 is primarily governed by sequence and structural features confined to the immediate initiation region, whereas inclusion of downstream coding sequences may introduce compensatory or antagonistic folding interactions that weaken the predictive power of global mRNA folding metrics.

Together, these results demonstrate that mRNA secondary structure within the N-terminal coding context is a major contributor among the factors examined to fusion-length-dependent expression variability, particularly when a sufficiently long coding sequence context is considered. The sharp improvement in correlation beyond approximately +90 nucleotides provides a mechanistic explanation for why N-terminal fusions of around 90 bp are sufficient to restore accurate regulatory sequence characterization, whereas shorter fusions fail to capture critical gene-specific folding constraints.

### 3.5 N-terminal fusion reveals latent regulatory constraints encoded within N-terminal coding sequences

To explore the boundary of the mechanistic framework described above, we further examined two genes, *b2943* and *b3952* (Supplementary Fig. 10), which exhibited atypical expression behaviors upon N-terminal fusion in our previous study [18]. Unlike the four target genes analyzed above, both genes displayed inconsistent or non-intuitive changes in regulatory sequence characterization despite fusing N-terminal sequences or increasing fusion lengths.

**Fig. 7:**
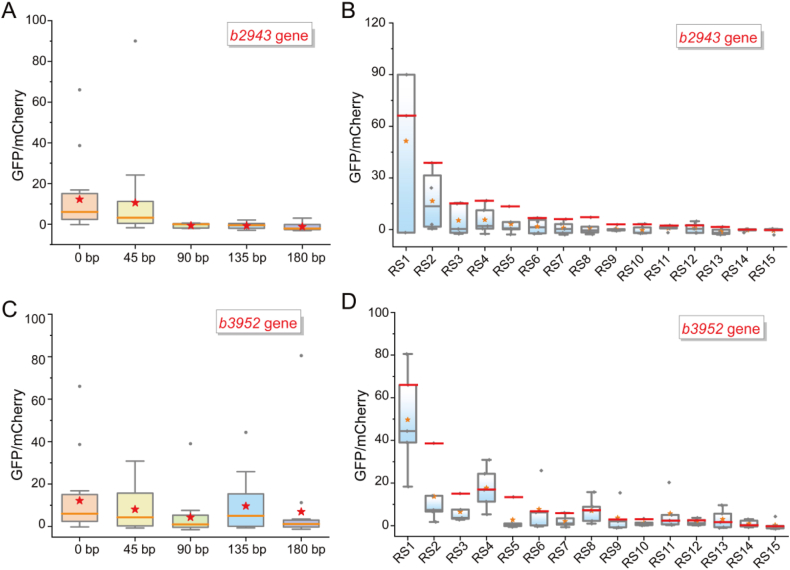
Effects of N-terminal fusion for *b2943* and *b3952* on regulatory sequence strengths. Expression strengths distribution across increasing N-terminal fusion lengths for *b2943* (A) and *b3952* (C). Expression strengths distribution under the control of each regulatory sequence following fusion with N-terminal coding sequence from *b2943* (B) and *b3952* (D). The box represents the data point between first quartile and third quartile, the central line indicates the median, and the star represents the mean. The red line in (B) or (D) denotes the fluorescence of unfused GFP.

At the protein expression level, N-terminal fusion failed to substantially enhance fluorescence output for both genes (Fig. 7). Instead, under most regulatory sequences, the fused constructs exhibited lower GFP/mCherry values than the unfused GFP controls (Fig. 7B–D). Moreover, as fusion length increased, fluorescence intensities driven by different regulatory sequences progressively converged toward narrower ranges. For *b2943*, this convergence occurred toward uniformly low expression levels across regulatory sequences, whereas for *b3952*, pronounced convergence was observed at fusion lengths of 90 bp and 180 bp.

For *b2943*, predicted translation initiation rates remained comparable to or lower than those of unfused *gfp* across fusion constructs, consistent with its overall poor expression (Supplementary Fig. 11). In contrast, predicted translation initiation rates for *b3952* were consistently higher than those of unfused *gfp* across fusion constructs. However, this increase did not translate into a corresponding enhancement in protein expression, indicating a decoupling between initiation efficiency and expression output.

When *b2943* and *b3952* were considered together, correlations between fluorescence intensity and mRNA MFE values exhibited weak, moderate, or even strong negative trends (Supplementary Fig. 12), in stark contrast to the predominantly positive correlations observed for the four target genes analyzed above. This inversion suggests that additional, gene-specific constraints—beyond general effects of translation initiation and N-terminal RNA folding—may dominate expression outcomes for these sequences.

Importantly, previous studies have reported the presence of transcription factor binding sites within the N-terminal coding sequence of *b2*943 that exert repressive effects on gene expression [47], providing independent evidence that strong *cis*-encoded regulatory constraints are embedded within its N-terminal sequence. Although no analogous regulatory elements have yet been identified for *b3952*, its expression behavior similarly deviates from predictions based on translation initiation efficiency or mRNA secondary structure alone, implying the involvement of additional gene-specific, post-initiation regulatory processes.

Together, these results indicate that while mRNA secondary structure within the N-terminal coding sequence is a major contributor to fusion-length-dependent expression variability in many tested contexts, whereas additional regulatory constraints encoded within N-terminal coding sequences can override or obscure this relationship in specific cases. These boundary cases highlight that N-terminal fusion reporters not only improve the fidelity of regulatory sequence characterization, but also function as sensitive probes for uncovering latent, gene-specific regulatory features that remain inaccessible to conventional unfused reporter systems.

## 4 Discussion

In our previous study, the 180 bp N-terminal fusion was used as an effective empirical strategy to improve compatibility analysis between regulatory sequences and target genes. In this study, we systematically evaluated how N-terminal coding sequence length shapes the accuracy of reporter-based regulatory-sequence characterization in target-gene-specific coding contexts. By comparing unfused reporters with a series of N-terminal fusion constructs of defined lengths across multiple genes and regulatory contexts, we demonstrate that the commonly adopted 180 bp fusion strategy, while effective, is not strictly required to achieve reliable regulatory characterization. Although the very proximal region surrounding the start codon is known to play a critical role in translation initiation, our results demonstrate that short N-terminal fusions (45 bp) failed to restore correspondence between reporter fluorescence and target protein activity for context-sensitive genes, with correlation coefficients remaining low (R^2^ < 0.2 for *lacZ*, Fig. 2). Instead, extending the fusion length to approximately 90 bp was sufficient in most tested contexts to preserve the correspondence between reporter output and target protein function, achieving strong or very strong positive correlations (mean R^2^ > 0.75) that approached those obtained with 180 bp fusions across multiple genes and growth stages. To further examine whether this practical threshold could be extended to additional sequence contexts, we performed an extended validation using two genes that were not included in the original four-gene dataset: the *gdhA* gene (encoding glutamate dehydrogenase, GDH) from *E. coli* and the *icd* gene (encoding isocitrate dehydrogenase) from *Pseudomonas putida*. The 90 bp and 180 bp N-terminal coding sequences of these genes were fused to GFP and tested under eight promoter-RBS combinations with different strengths (Supplementary Fig. 13, Supplementary Data). The resulting fluorescence outputs were compared with the corresponding enzyme activities. Consistent with the main dataset, the 90 bp fusion showed correlations with enzyme activity comparable to those obtained with the 180 bp fusion (Supplementary Fig. 13). In particular, for the context-sensitive *gdhA* case, N-terminal fusion substantially improved the correlation between normalized GFP fluorescence and GDH enzyme activity at 6 h, with R^2^ values reaching 0.96-0.98 for the fusion reporters compared with 0.62 for the unfused GFP reporter. Together, these findings support the view that, in many tested contexts, a 90 bp N-terminal fusion can capture most of the relevant target-dependent coding-context information while reducing the sequence-length burden.

Using a same set of regulatory sequences, we further reveal pronounced gene-dependent context effects that govern both the magnitude and nature of fusion-dependent expression changes. Firstly, the extent to which N-terminal fusion improves regulatory characterization varies across genes. For some genes, such as *lacZ* and *icd*, unfused reporters exhibited poor correlation between reporter fluorescence and target protein activity (Fig. 2C), whereas N-terminal fusion substantially improved this relationship. By contrast, other genes, such as *bfp*, displayed relatively high correspondence regardless of fusion strategy. Secondly, the impact of N-terminal fusions on the strength distribution of regulatory sequences is also gene-dependent. For *lacZ* and *icd*, N-terminal fusions substantially expanded the observable dynamic range of regulatory sequences (Fig. 3), with strong regulatory sequences exhibiting greater variability across fusion contexts than weak ones. In contrast, regulatory sequences driving the expression of *zwf* showed comparatively stable behavior, with limited redistribution of apparent strength upon fusion. This behavior provides a mechanistic basis for the frequently observed lack of portability of regulatory elements: “strength” is not an intrinsic property of a promoter/RBS alone, but an emergent outcome of its interaction with downstream coding context. Importantly, transcript-level differences across the same construct sets were noticeably more compressed than protein-level differences, and the correspondence between mRNA abundance and fluorescence was regulatory sequence dependent, ranging from weak to moderate (Supplementary Fig. 6). Together, these observations indicate that post-transcriptional mechanisms might play a major role in the fusion-dependent reshaping of regulatory outputs.

Mechanistically, our analyses point to sequence features within the N-terminal coding context as major contributors to fusion-dependent expression variability. Model-based predictions suggest that changes in transcription initiation and translation initiation are unlikely to be the main drivers: for most constructs, TSS positions and predicted initiation rates remain stable across fusion lengths, and predicted translation initiation rates do not systematically track the length-dependent expression shifts. In contrast, mRNA secondary structure showed a closer association with fusion-length-dependent expression changes than the predicted transcription or translation initiation features, with less stable predicted folding (higher, i.e., less negative, MFE) generally associated with increased protein output. A focused synonymous mutation analysis under RS8 provided additional, but qualified, support for this interpretation (Supplementary Figs. 14 and 15). Synonymous variants in the N-terminal coding region altered reporter fluorescence despite encoding identical peptide sequences, supporting the importance of sequence-level features in this region. However, these changes were not consistently predicted by MFE values alone, indicating that mRNA secondary structure should be interpreted as a major contributor among the factors examined rather than the sole determinant. In the native fusion-length series, these effects were most pronounced when mRNA secondary structure was evaluated over an extended N-terminal window rather than being confined to the immediate initiation region (Fig. 6B), underscoring the functional relevance of downstream coding sequence context. This trend was further supported by 1 bp resolution RNAfold scans across the first 180 bp region (Supplementary Fig. 16). Although the window position yielding the maximum correlation was regulatory-sequence-dependent, 11 out of 15 regulatory sequences achieved at least 80% of their maximal |r| when the analysis window extended beyond approximately 90 bp. To identify potential local structural transition points, we first visualized 1-bp resolution MFE profiles for the four standard target genes (*lacZ*, *icd*, *zwf* and *bfp*) across all 15 regulatory-sequence contexts (Supplementary Fig. 17). These profiles revealed gene- and regulatory-sequence-dependent local MFE decreases within the first 180 bp. We further analyzed the positions showing the maximum 1 bp ΔMFE drop across the four target genes under the control of 15 regulatory sequences (Supplementary Fig. 18). These positions were not restricted to a single nucleotide site, but were frequently observed within the approximately 45-90 bp region, suggesting that this interval contains recurrent local folding transitions. Thus, the 90 bp fusion length represents the shortest experimentally tested effective window that captures most of the informative N-terminal structural context, rather than an exact nucleotide-level optimum. Together, these findings explain why regulatory sequences characterized using a single unfused reporter often fail to generalize across diverse target genes, as the interaction between regulatory sequences and gene-specific N-terminal coding features fundamentally reshapes expression outcomes.

Importantly, we also identified boundary cases that deviate from these dominant trends. For *b2943* and *b3952*, longer fusions did not increase protein output but instead compressed the regulatory dynamic range, with weak or even negative structure-expression relationships. To further examine these boundary cases, we applied the same 1 bp resolution MFE scan and ΔMFE-drop analysis to *b2943* and *b3952* (Supplementary Figs. 19–21). These analyses did not reveal any unique inhibitory structural motif or exceptionally stable local stem that clearly distinguished these genes from the standard cases. Thus, the boundary behavior of *b2943* and *b3952* is unlikely to be explained by MFE profiles alone. Instead, these behaviors suggest that gene-specific, *cis*-encoded constraints can override generic N-terminal context effects (*b2943* has been implicated to contain repressive features [47], whereas the basis for *b3952* remains unclear despite a decoupling between predicted initiation efficiency and measured output). Collectively, these cases show that N-terminal fusion reporters not only improve characterization fidelity in typical settings but can also reveal latent gene-specific regulatory constraints that are not apparent with unfused reporters. Moreover, the boundary cases further emphasize the need to integrate high-resolution MFE analysis with additional sequence and expression features before developing a broadly predictive model for gene-specific fusion-length design.

By defining a practical and context-preserving N-terminal fusion strategy, our results provide a mechanistically informed basis for optimizing regulatory sequence characterization while enabling scalable, high-throughput applications. By reducing fusion length without substantially compromising characterization accuracy in most tested contexts, truncated N-terminal fusions enable more compact library designs that are compatible with high-throughput platforms such as MPRAs and FACS-seq. This improvement alleviates constraints imposed by finite oligonucleotide-pool lengths and reduces synthesis costs, thereby expanding the diversity of regulatory sequences that can be interrogated. Nevertheless, the choice of fusion length should be guided by the intended application. For most tested contexts, the 90 bp fusion can serve as a practical default starting point for scalable regulatory-sequence profiling, whereas high-resolution mRNA design tools can be used to identify genes that may require extended N-terminal context. Longer fusions, such as 135 bp or 180 bp, may be preferred when maximal reporter-target correlation is required or when gene-specific downstream structural features or predicted structural transitions beyond 90 bp are suspected. Moreover, expression datasets generated using such context-preserving reporter designs may provide higher-quality inputs for data-driven modeling approaches, including machine learning [8,48], and support more predictable multi-gene expression and pathway optimization.

## CRediT authorship contribution statement

**Sen Yang:** Data curation, Formal analysis, Investigation. **Ye Liu:** Conceptualization, Data curation, Formal analysis, Funding acquisition, Methodology, Writing – original draft, Writing – review & editing. **Anqi Li:** Investigation. **Shenhui Wang:** Software. **Jiawen Cui:** Investigation. **Huanhuan Liu:** Supervision. **Meng Wang:** Funding acquisition, Supervision, Writing – review & editing. **Yufeng Mao:** Conceptualization, Formal analysis, Funding acquisition, Writing – review & editing.

## Declaration of competing interest

The authors declare that they have no known competing financial interests or personal relationships that could have appeared to influence the work reported in this paper.
