# Supplementary material for: N-terminal fusion length: The key to reliable and context-preserving regulatory sequence characterization
Source: Synth Syst Biotechnol. 2026 Jul 22;16:107–18. doi: 10.1016/j.synbio.2026.05.020 (PMC13427500; doi:10.1016/j.synbio.2026.05.020)
Supplement: Multimedia component 1 [file mmc1.pdf]

Supplementary information for

N-terminal fusion length: The key to reliable and context-preserving regulatory sequence characterization

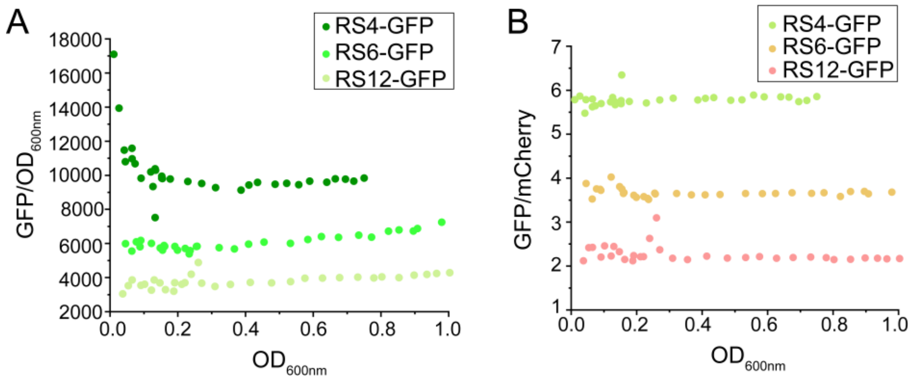

Supplementary Fig. 1. Comparison of different fluorescence normalization methods. Cultured cells with three different GFP expression levels were serially diluted to generate a range of cell densities. (A) GFP/OD_{600nm} normalization distribution of the diluted cells. (B) GFP/mCherry normalization distribution of the diluted cells.

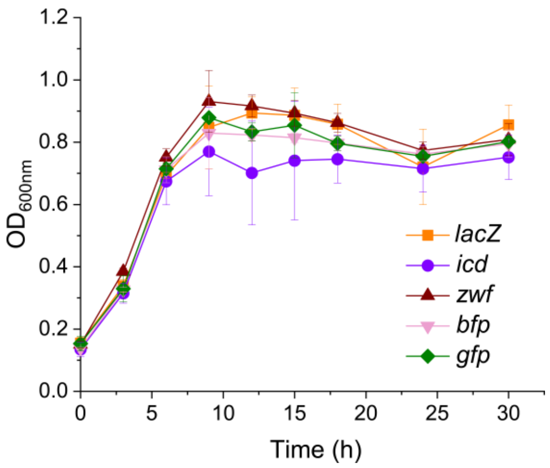

Supplementary Fig. 2. Growth curves of strains overexpressing different genes.

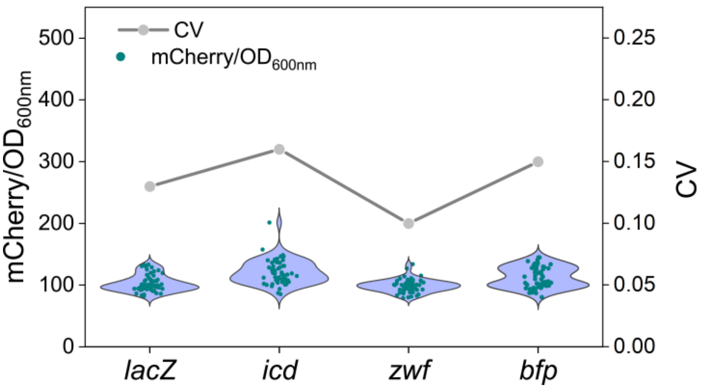

Supplementary Fig. 3. mCherry expression levels and the corresponding coefficients of variation (CV) for different GFP fusion constructs at 6 h of growth.

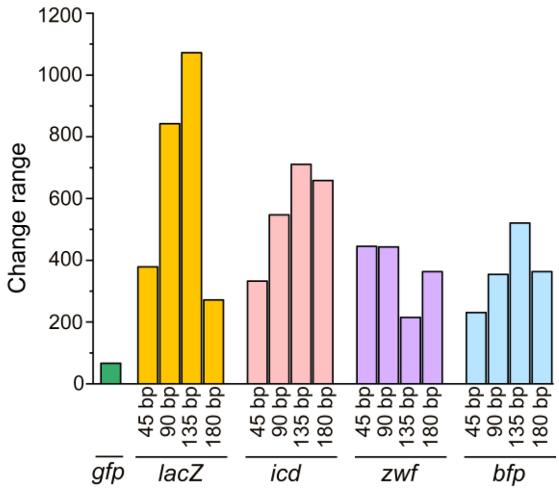

Supplementary Fig. 4. Change range (maximum minus minimum) of normalized GFP/mCherry expression strengths for all constructs after fusion with N-terminal coding sequences from four target genes (*lacZ*, *icd*, *zwf* and *bfp*).

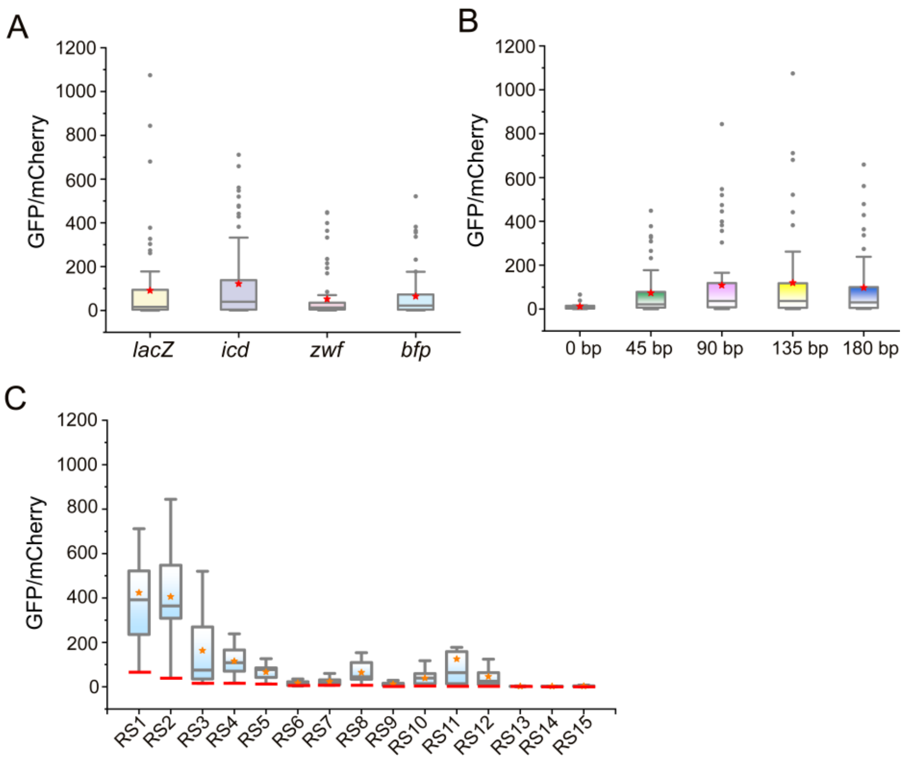

Supplementary Fig. 5. Summary distribution of normalized expression strengths for all constructs after fusion with N-terminal coding sequences from four target genes (*lacZ*, *icd*, *zwf* and *bfp*) at 6 h of growth. (A) Gene-type-dependent distribution of expression strengths. (B) Fusion-length-dependent distribution of expression strengths. (C) Regulatory-sequence-dependent distribution of expression strengths. The box represents the data point between first quartile and third quartile, the central line indicates the median, and the star represents the mean. The red line in (C) denotes the fluorescence of unfused GFP.

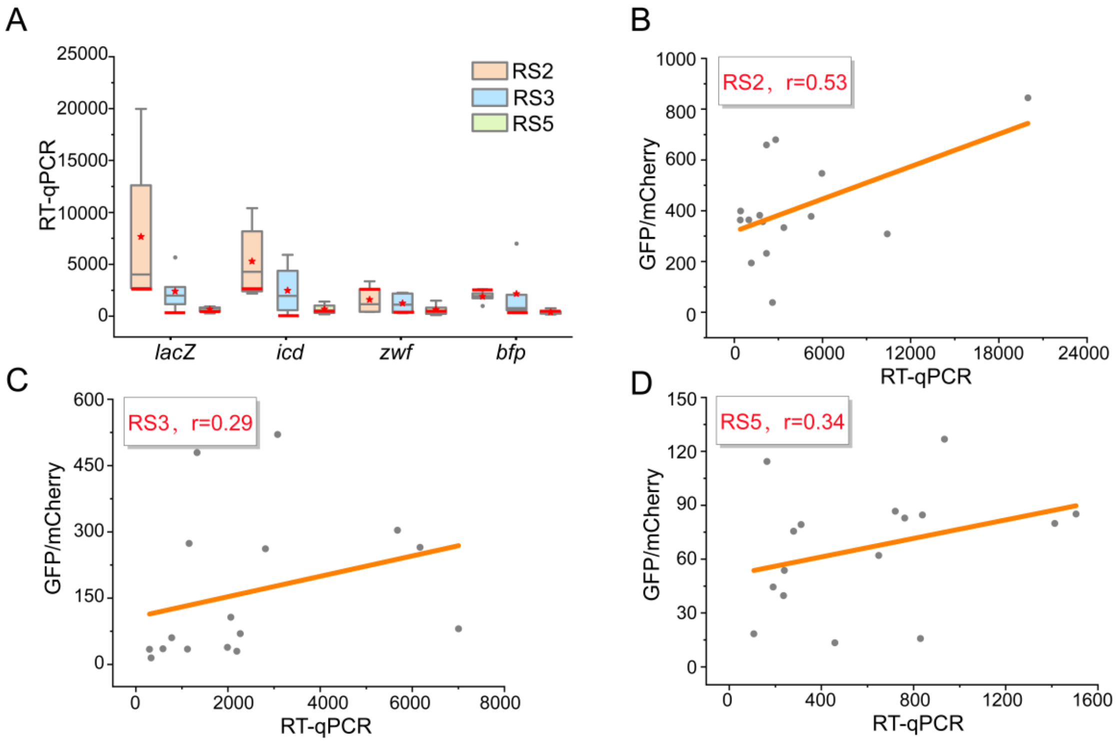

Supplementary Fig. 6. Transcription levels of genes encoding different GFP fusion proteins. (A) Changes in transcription level driven by RS2, RS3 and RS5 following fusion with N-terminal coding sequences of varying lengths from the four target genes. Correlation between GFP fluorescence and transcription levels for different GFP fusion constructs driven by RS2 (B), RS3 (C) and RS5 (D).

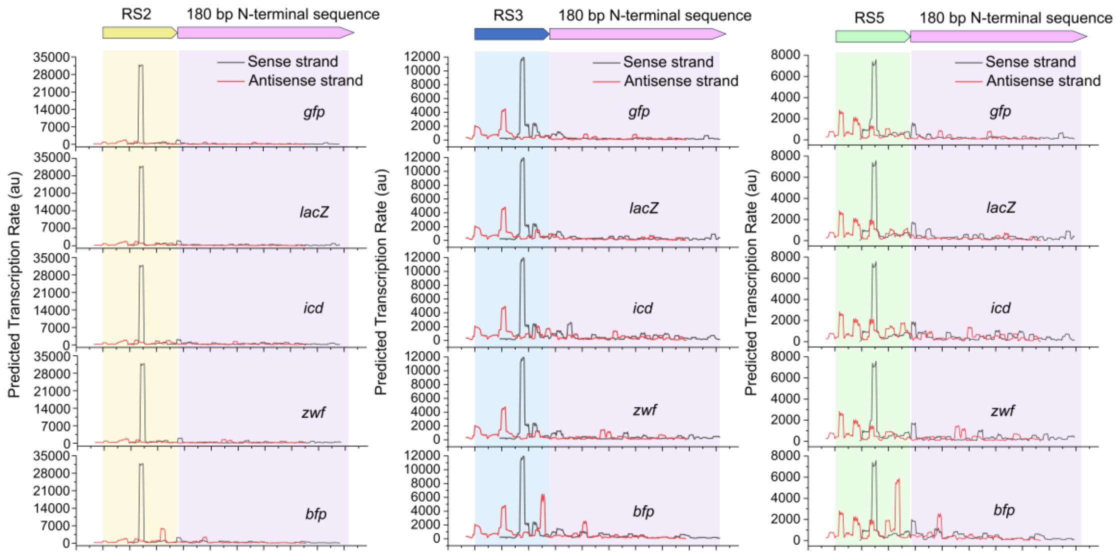

Supplementary Fig. 7. The predicted transcriptional profiles for the first 180 bp of different target genes driven by RS2, RS3 and RS5. The black line represents the transcription start site and its transcription rate predicted on the sense strand, while the red line represents those predicted on the antisense strand.

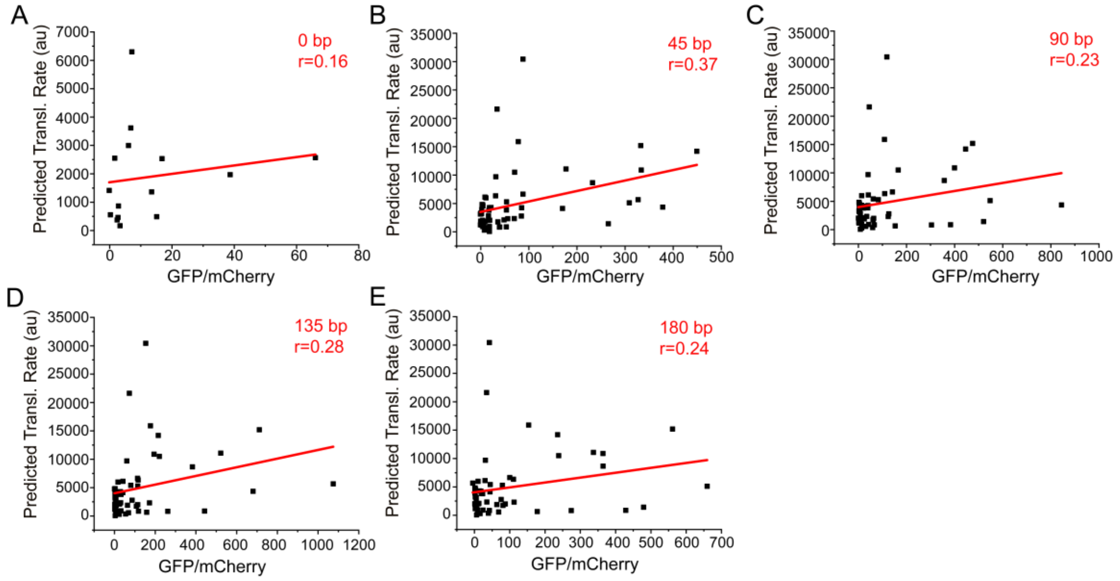

Supplementary Fig. 8. Correlation between predicted translation initiation rate, calculated using the RBS Calculator v2.1, and GFP fluorescence for different GFP fusion constructs (at 6 h of growth) across increasing N-terminal fusion lengths (A-E). For each target gene, the predicted translation initiation rate remained essentially constant across fusion lengths.

A

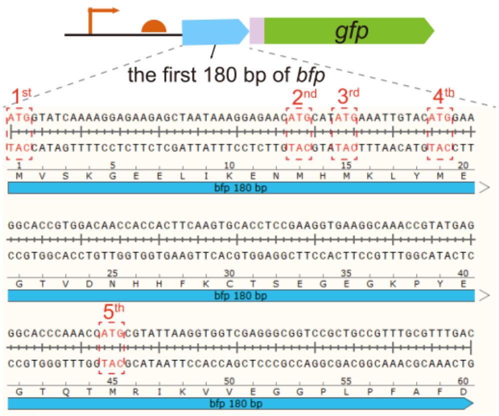

B

Regulatory sequence	1st ATG	2nd ATG	3rd ATG	4th ATG	5th ATG
RS1	11092.20	3610.89	3496.27	24.32	8.37
RS2	8661.58	971.23	1250.03	9.43	8.37
RS3	1736.35	1891.76	935.93	5.41	8.37
RS4	15891.84	5137.72	3436.06	8.61	8.37
RS5	5286.24	611.34	362.08	2.73	8.37
RS6	3801.19	1456.23	1365.91	10.08	8.37
RS7	5998.60	296.40	427.02	1.50	8.37
RS8	21621.85	413.87	227.48	1.17	8.37
RS9	307.53	1350.68	825.05	6.22	8.37
RS10	584.31	1752.43	948.57	7.16	8.37
RS11	2007.48	830.21	3253.36	23.68	8.37
RS12	1892.72	10844.78	5536.56	41.77	8.37
RS13	1182.07	5191.64	3374.76	25.46	8.37
RS14	3922.85	1516.43	1925.54	11.39	8.37
RS15	3190.72	357.78	771.26	5.82	8.37

Supplementary Fig. 9. Additional translation initiation events in the *bfp* gene. (A) In-frame start codons within the first 180 bp of the *bfp* gene. (B) Predicted translation initiation rate for each in-frame start codon under the control of 15 regulator sequences, with the maximum rate marked in red.

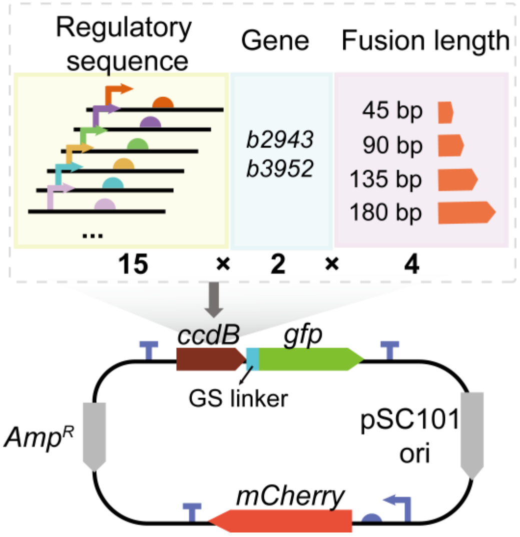

Supplementary Fig. 10. Design of GFP fusion library containing N-terminal coding sequences of varying lengths from *b2943* and *b3952*

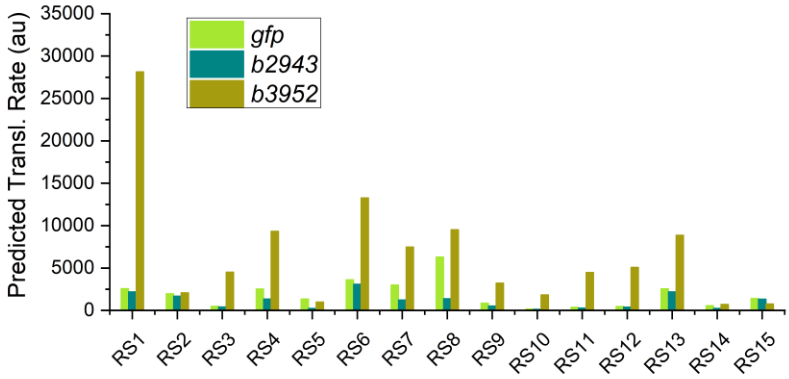

Supplementary Fig. 11. Translation initiation rates were predicted for each GFP fusion construct containing N-terminal coding sequences from *b2943* and *b3952* using the RBS Calculator v2.1.

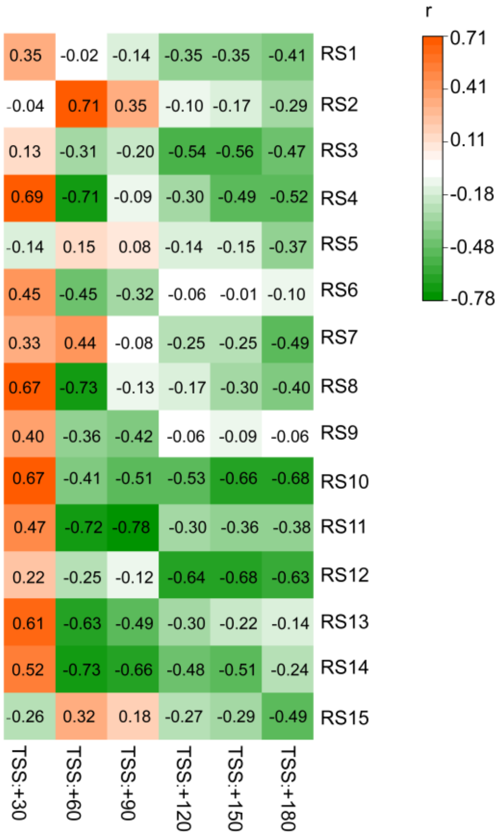

Supplementary Fig. 12. Correlation between normalized GFP/mCherry fluorescence (at 6 h of growth) and the predicted minimum free energy (MFE) of mRNA folding for GFP fusion constructs containing N-terminal coding sequences from *b2943* and *b3952*.

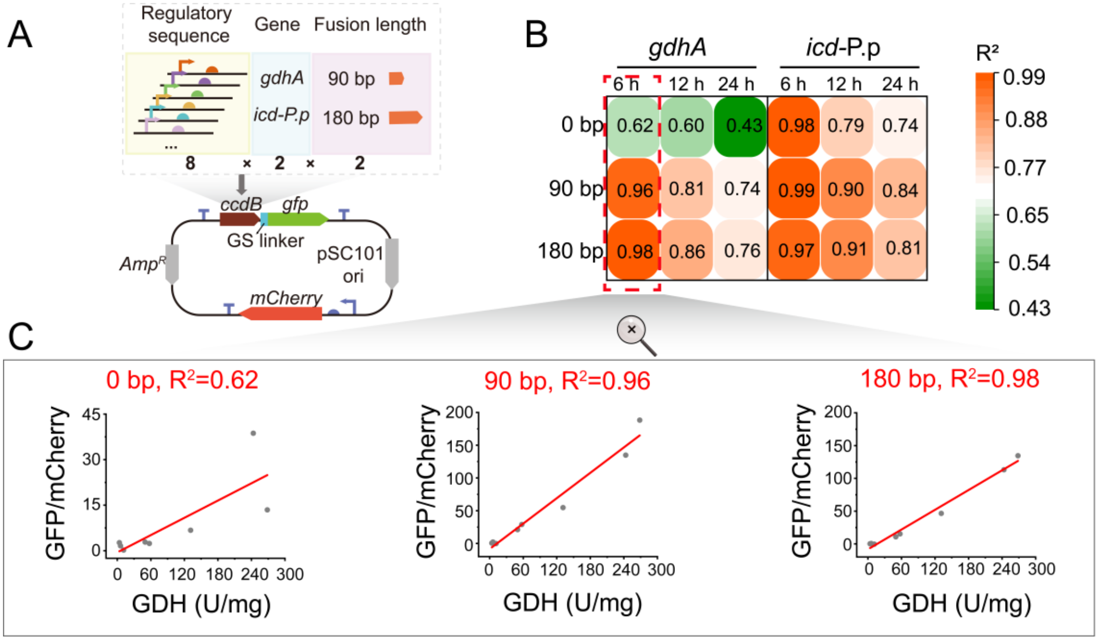

Supplementary Fig. 13. Extended validation of regulatory sequence characterization using N-terminal fusion strategies in additional genes. (A) Design of GFP reporter library containing N-terminal coding sequences of varying lengths from *gdhA* and *icd-P.p* (icd gene from *Pseudomonas putida* KT2440), each driven by 8 pre-characterized regulatory sequences. (B) Correlation (R^2) analysis between normalized GFP/mCherry fluorescence and corresponding enzyme activities across fusion constructs. (C) Detailed correlation analysis between normalized GFP/mCherry fluorescence and GDH enzyme activity for *gdhA* fusion constructs with varying N-terminal fusion lengths at 6 h of growth.

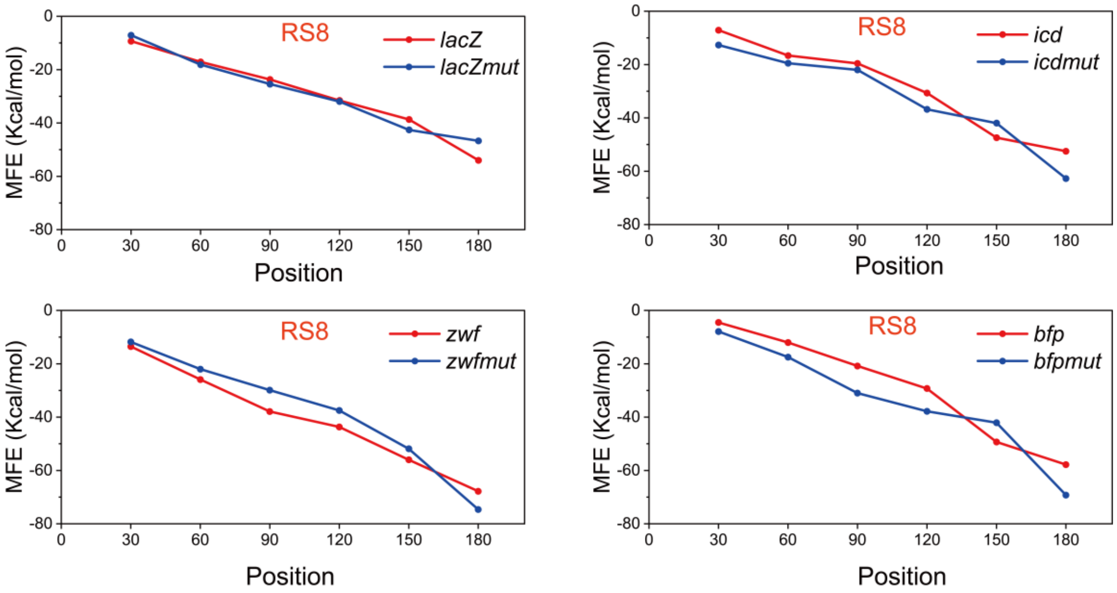

Supplementary Fig. 14. Comparison of predicted MFEs between GFP fusion constructs containing the native 180 bp N-terminal coding sequences (*lacZ*, *icd*, *zwf* and *bfp*) and their synonymous mutants (*lacZmut*, *icdmut*, *zwfmut* and *bfpmut*) under RS8.

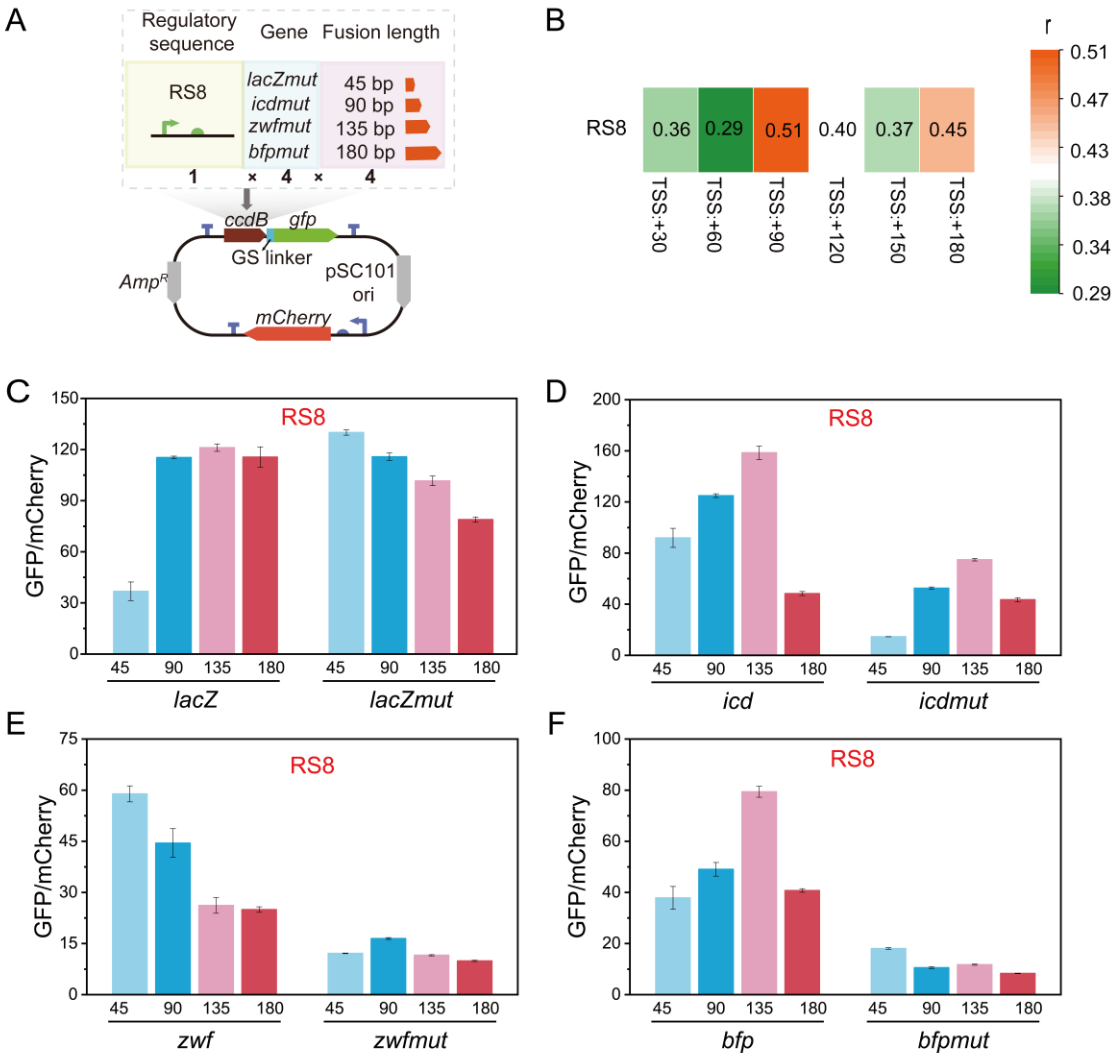

Supplementary Fig. 15. Fluorescence analysis of GFP fusion constructs containing synonymous mutant N-terminal coding sequences under the control of RS8. (A) Design of GFP fusion library containing N-terminal coding sequences of varying lengths from mutant genes (*lacZmut*, *icdmut*, *zwfmut* and *bfpmut*). (B) Correlation between normalized GFP/mCherry fluorescence (at 6 h of growth) and predicted MFE for GFP fusion constructs containing N-terminal coding sequences of varying lengths derived from four native genes (*lacZ*, *icd*, *zwf* and *bfp*) and their corresponding synonymous mutant genes (*lacZmut*, *icdmut*, *zwfmut* and *bfpmut*) under the control of RS8. (C-F) Fluorescence analysis of GFP fusion constructs carrying N-terminal coding sequences of varying lengths from each native gene and its corresponding synonymous mutant under RS8 regulation.

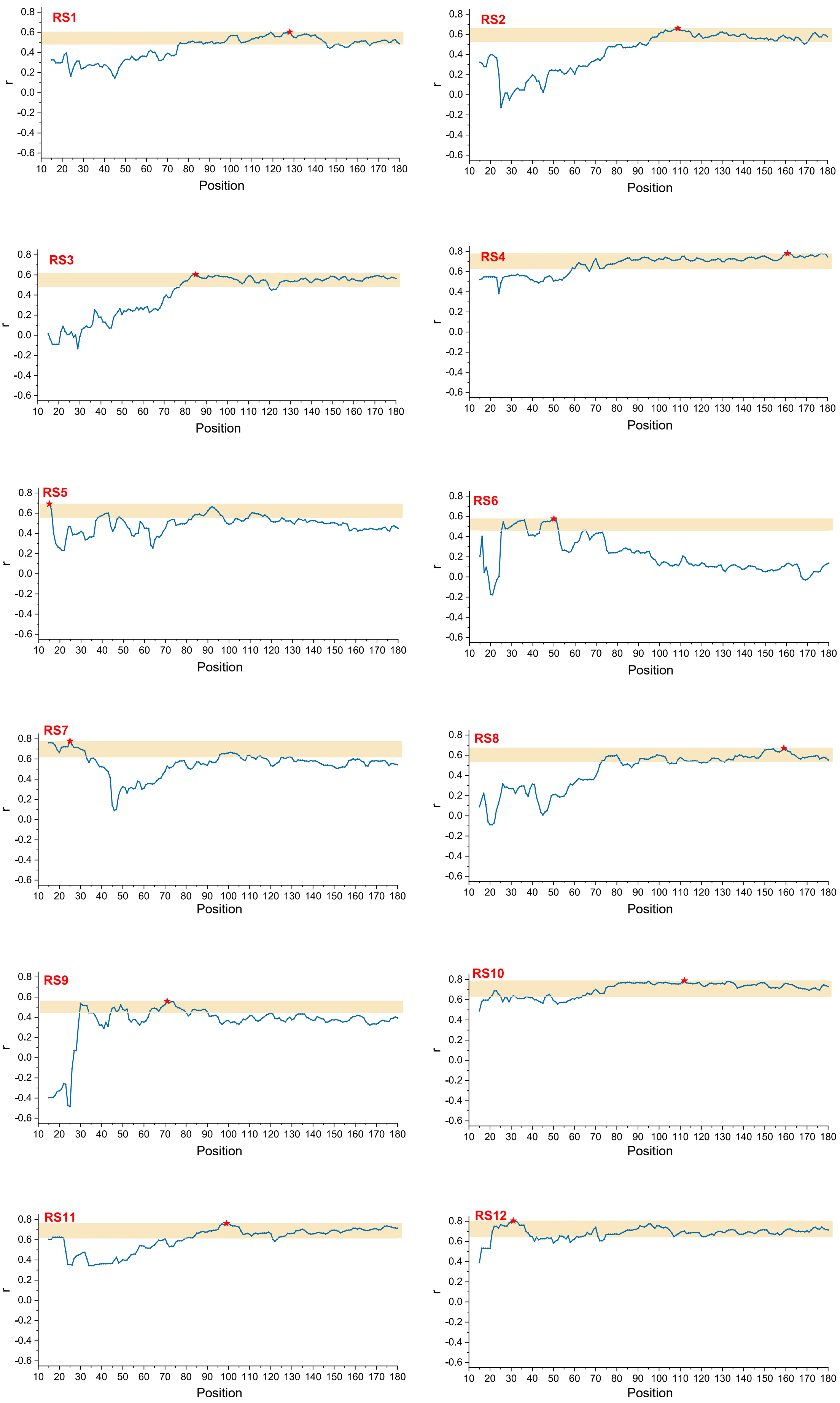
RS1
RS2
RS3
RS4
RS5
RS6
RS7
RS8
RS9
RS10
RS11
RS12
r
Position
0.8
0.6
0.4
0.2
0.0
-0.2
-0.4
-0.6
10 20 30 40 50 60 70 80 90 100 110 120 130 140 150 160 170 180

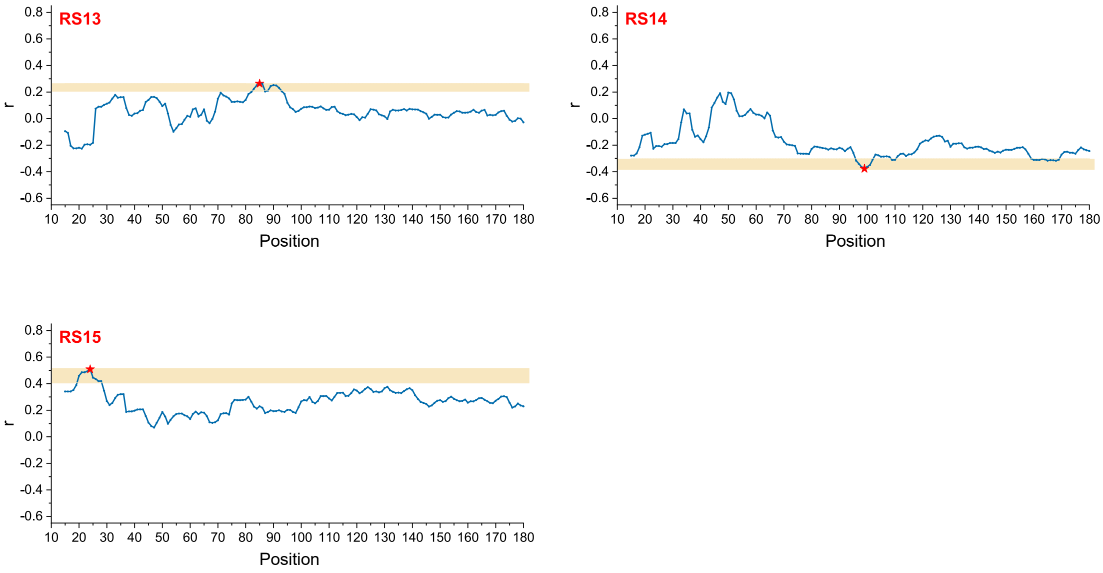

Supplementary Fig. 16. Detailed 1-bp resolution correlation analysis between normalized GFP/mCherry fluorescence and predicted MFE of mRNA folding for GFP fusion constructs containing N-terminal coding sequences from four target genes (*lacZ*, *icd*, *zwf* and *bfp*). Each panel represents results obtained under a distinct regulatory sequence context. The red star marks the position with the maximum correlation value, and the orange shaded region denotes the range corresponding to 80-100% of the maximum absolute correlation value (|r|).

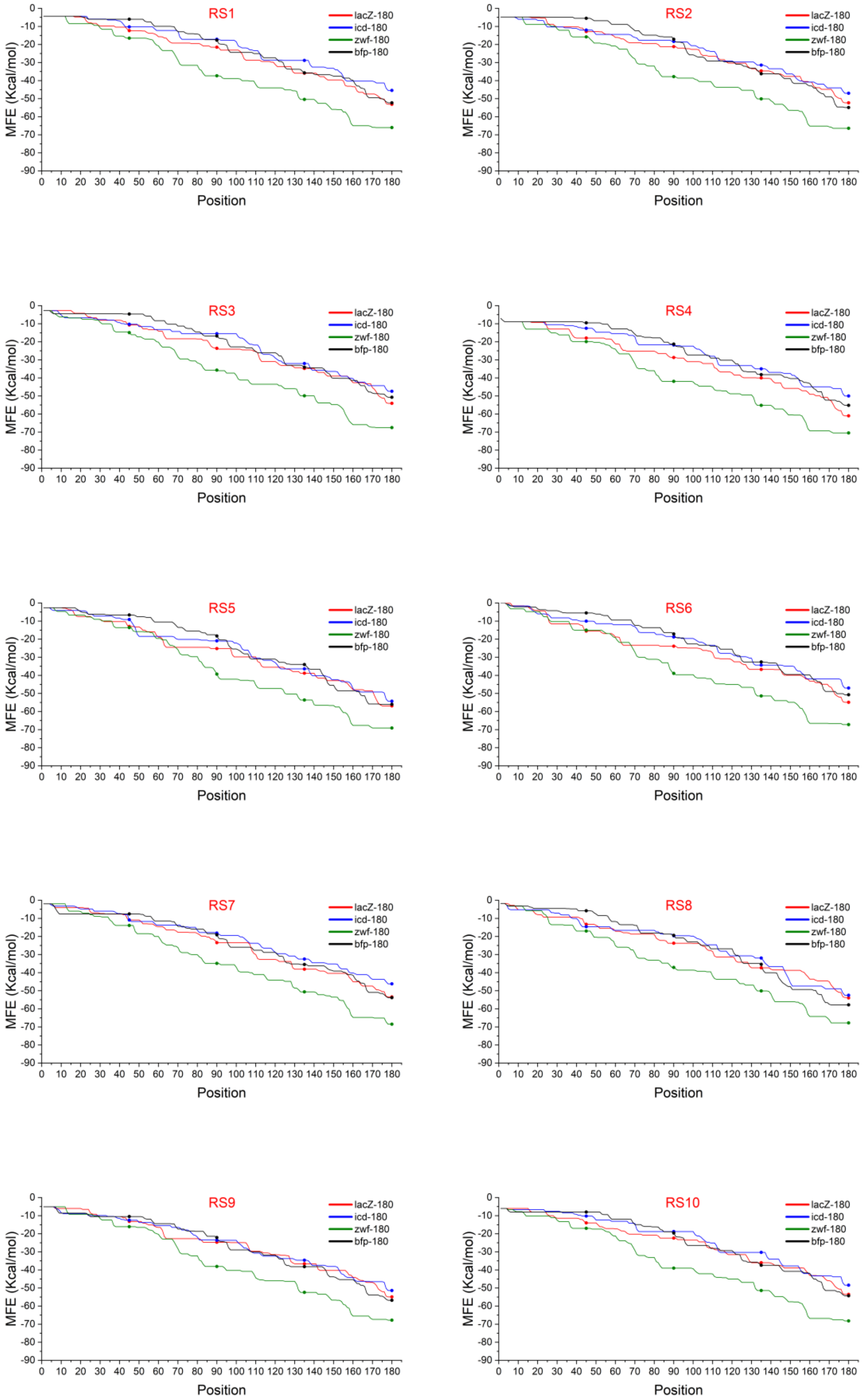
RS1
RS2
RS3
RS4
RS5
RS6
RS7
RS8
RS9
RS10
lacZ-180
icd-180
zwf-180
bfp-180
MFE (Kcal/mol)
Position

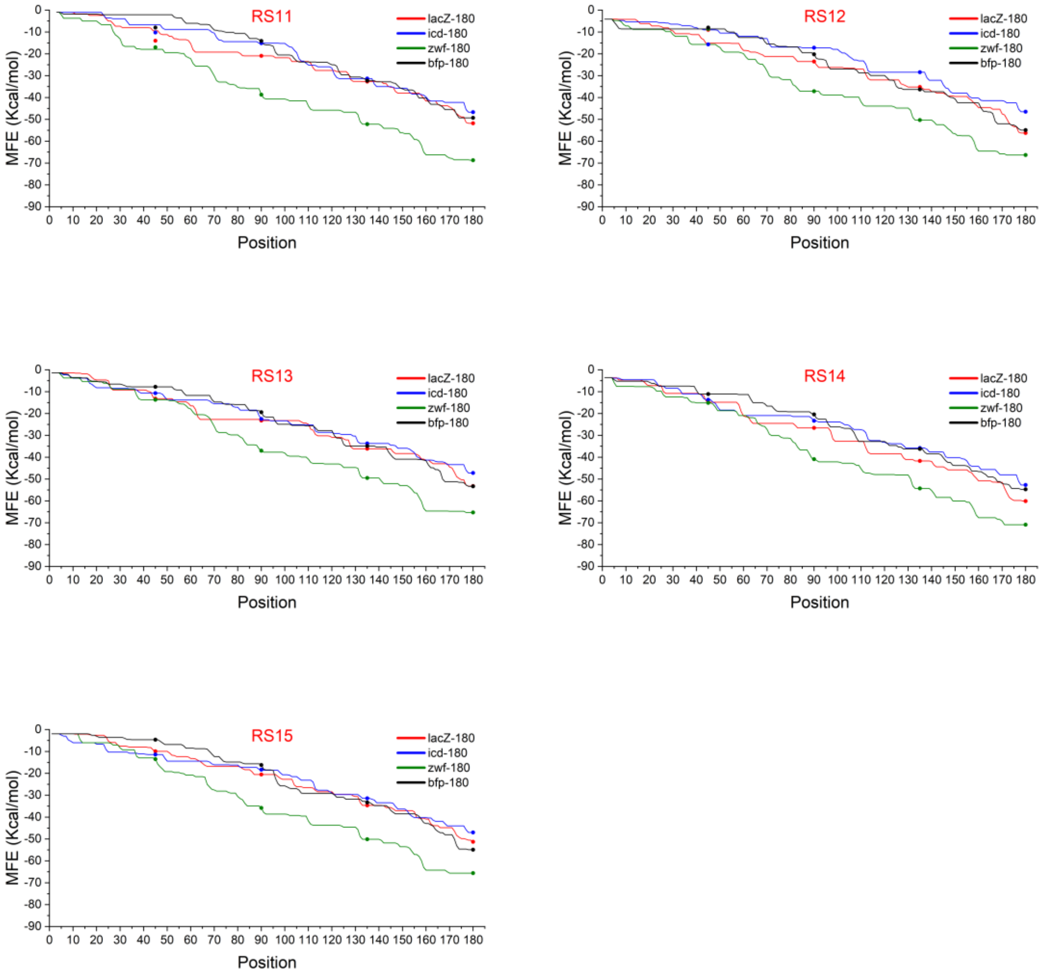

Supplementary Fig. 17. Detailed 1-bp resolution MFE analysis of mRNA folding for GFP fusion constructs containing the first 180 bp of N-terminal coding sequences from four target genes (*lacZ*, *icd*, *zwf* and *bfp*). Each panel represents a distinct regulatory sequence context. Dots indicate positions corresponding to 45, 90, 135, and 180 bp.

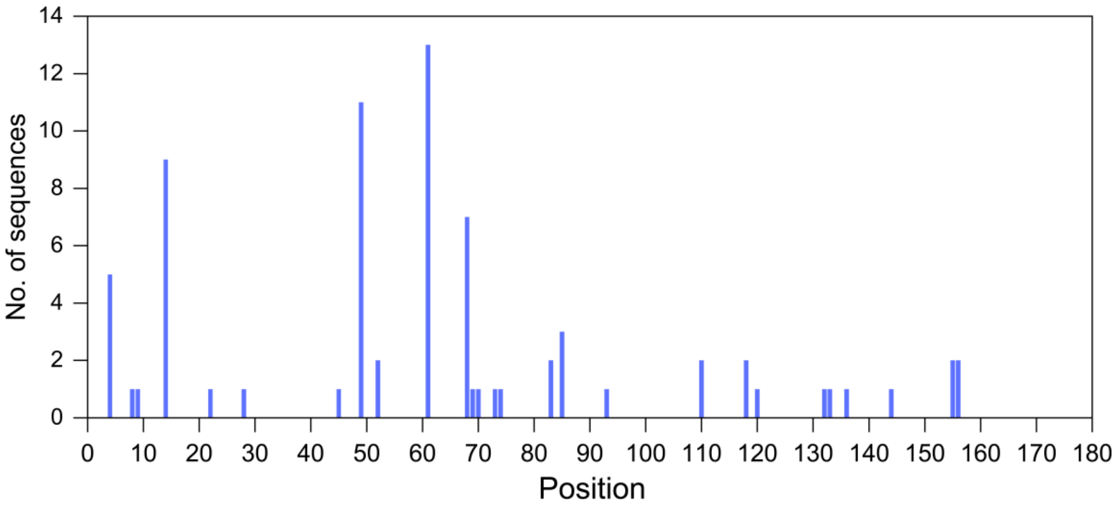

Supplementary Fig. 18. Distribution of positions showing the maximum 1-bp ΔMFE drop within the first 180 bp of N-terminal coding sequences. The x-axis indicates the position along the first 180 bp coding region, while the y-axis represents the number of sequences exhibiting the maximum ΔMFE drop at each position. Statistics were compiled from four target genes (*lacZ*, *icd*, *zwf* and *bfp*) under 15 distinct regulatory sequences.

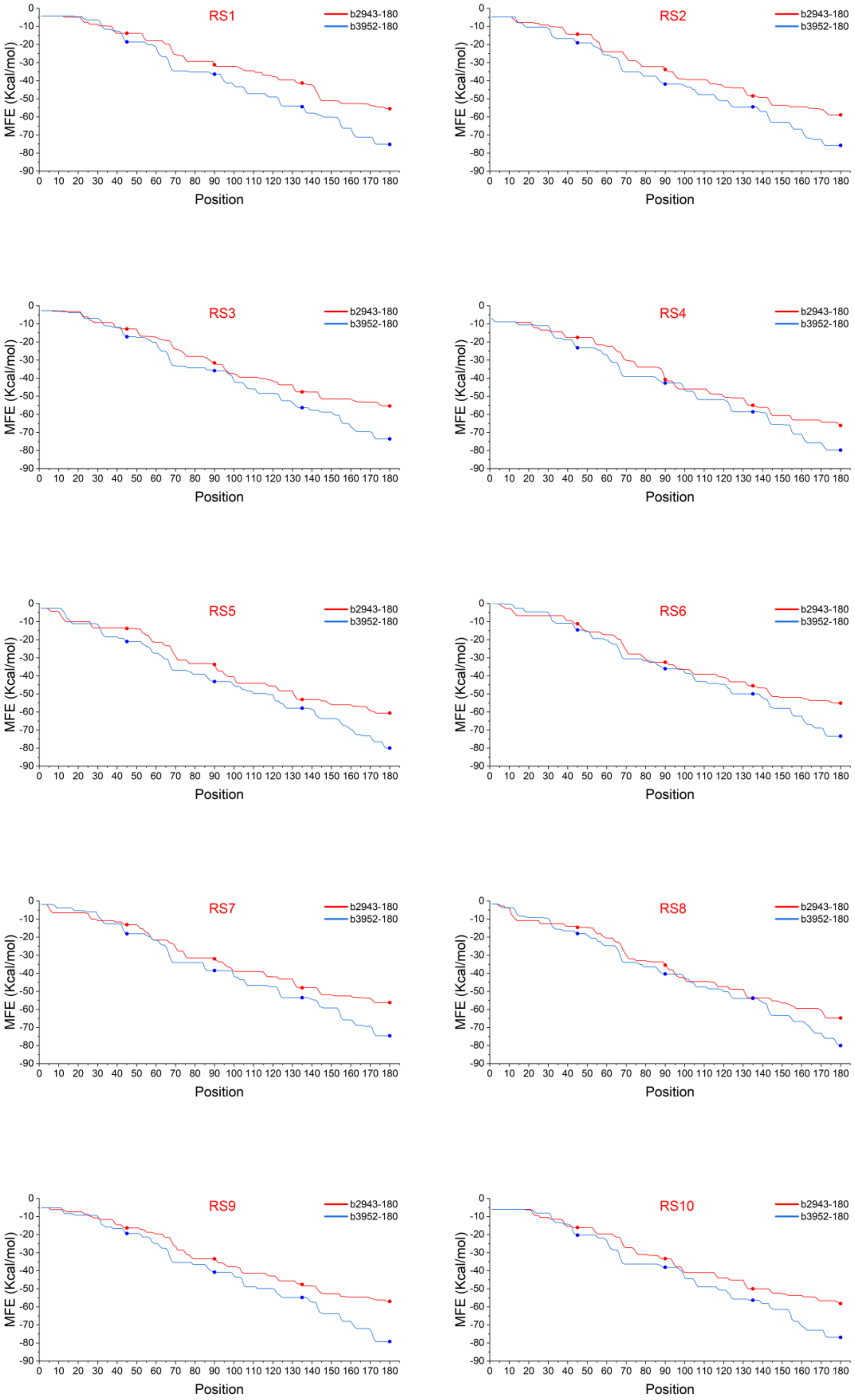
RS1
b2943-180
b3952-180
MFE (Kcal/mol)
0 -10 -20 -30 -40 -50 -60 -70 -80 -90
0 10 20 30 40 50 60 70 80 90 100 110 120 130 140 150 160 170 180
Position
RS2
b2943-180
b3952-180
MFE (Kcal/mol)
0 -10 -20 -30 -40 -50 -60 -70 -80 -90
0 10 20 30 40 50 60 70 80 90 100 110 120 130 140 150 160 170 180
Position
RS3
b2943-180
b3952-180
MFE (Kcal/mol)
0 -10 -20 -30 -40 -50 -60 -70 -80 -90
0 10 20 30 40 50 60 70 80 90 100 110 120 130 140 150 160 170 180
Position
RS4
b2943-180
b3952-180
MFE (Kcal/mol)
0 -10 -20 -30 -40 -50 -60 -70 -80 -90
0 10 20 30 40 50 60 70 80 90 100 110 120 130 140 150 160 170 180
Position
RS5
b2943-180
b3952-180
MFE (Kcal/mol)
0 -10 -20 -30 -40 -50 -60 -70 -80 -90
0 10 20 30 40 50 60 70 80 90 100 110 120 130 140 150 160 170 180
Position
RS6
b2943-180
b3952-180
MFE (Kcal/mol)
0 -10 -20 -30 -40 -50 -60 -70 -80 -90
0 10 20 30 40 50 60 70 80 90 100 110 120 130 140 150 160 170 180
Position
RS7
b2943-180
b3952-180
MFE (Kcal/mol)
0 -10 -20 -30 -40 -50 -60 -70 -80 -90
0 10 20 30 40 50 60 70 80 90 100 110 120 130 140 150 160 170 180
Position
RS8
b2943-180
b3952-180
MFE (Kcal/mol)
0 -10 -20 -30 -40 -50 -60 -70 -80 -90
0 10 20 30 40 50 60 70 80 90 100 110 120 130 140 150 160 170 180
Position
RS9
b2943-180
b3952-180
MFE (Kcal/mol)
0 -10 -20 -30 -40 -50 -60 -70 -80 -90
0 10 20 30 40 50 60 70 80 90 100 110 120 130 140 150 160 170 180
Position
RS10
b2943-180
b3952-180
MFE (Kcal/mol)
0 -10 -20 -30 -40 -50 -60 -70 -80 -90
0 10 20 30 40 50 60 70 80 90 100 110 120 130 140 150 160 170 180
Position

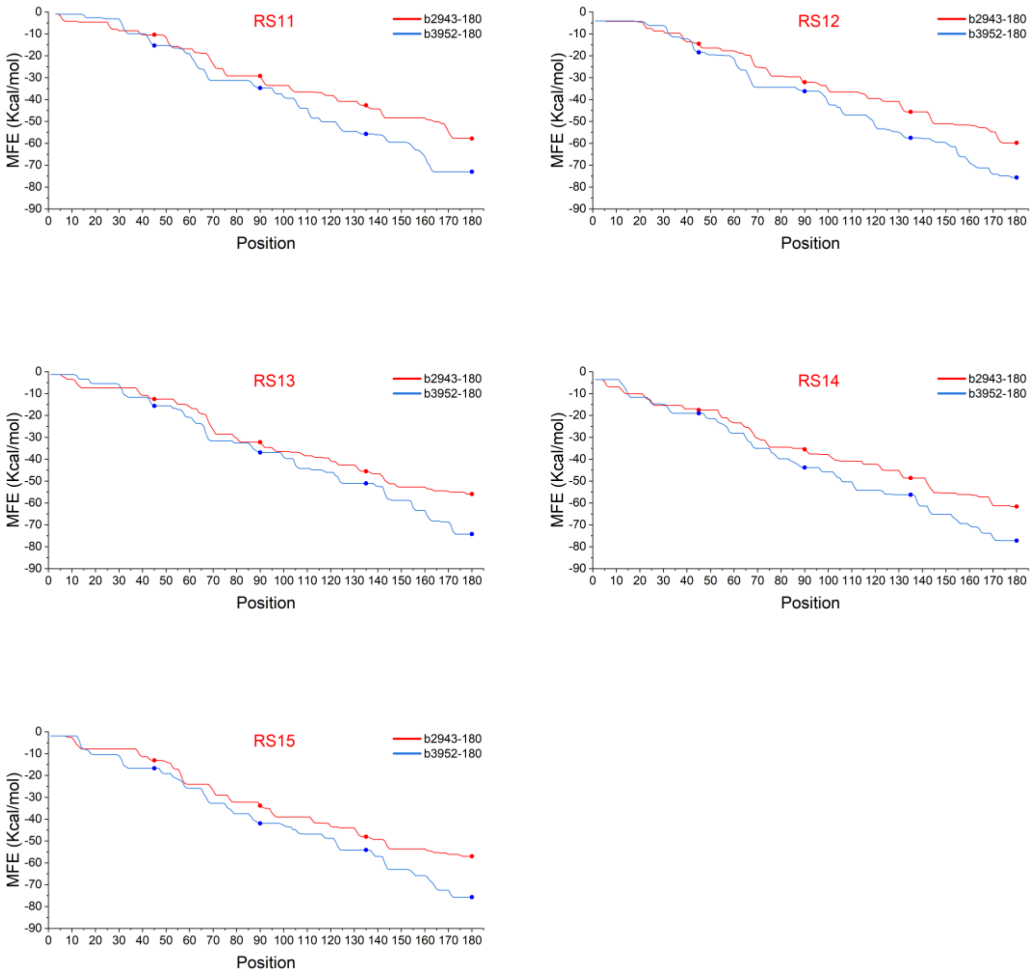

Supplementary Fig. 19. Detailed 1-bp resolution MFE analysis of mRNA folding for GFP fusion constructs containing the first 180 bp of N-terminal coding sequences from *b2943* and *b3952*. Each panel represents a distinct regulatory sequence context. Dots indicate positions corresponding to 45, 90, 135, and 180 bp.

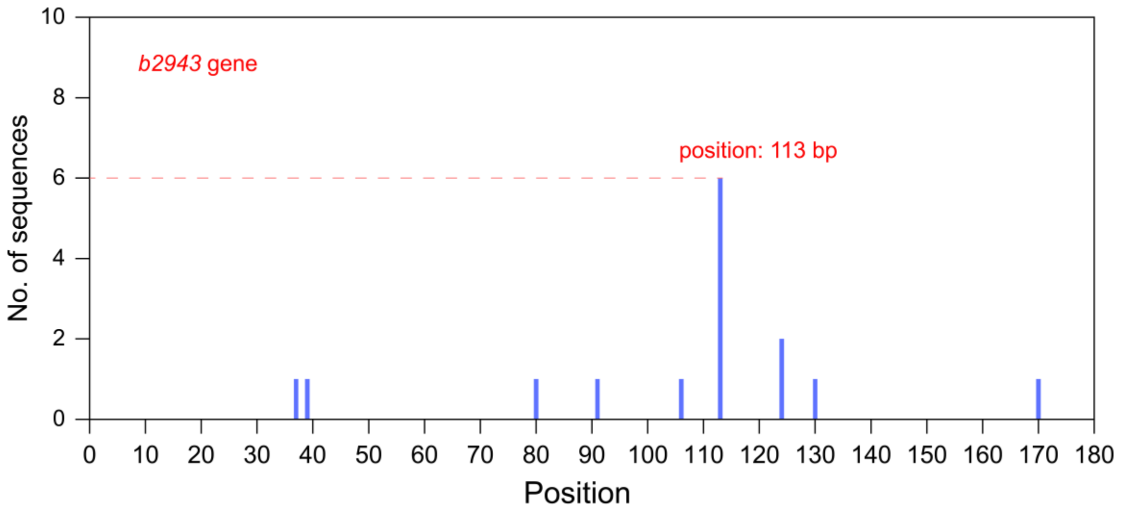

Supplementary Fig. 20. Distribution of positions showing the maximum 1-bp ΔMFE drop within the first 180 bp of the *b2943* N-terminal coding sequence under 15 distinct regulatory sequences.

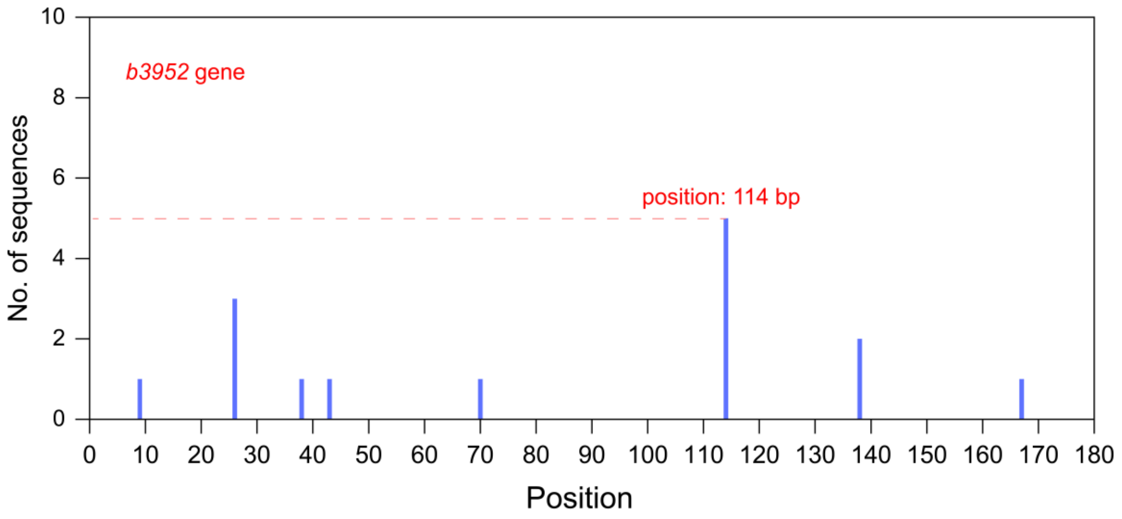

Supplementary Fig. 21. Distribution of positions showing the maximum 1-bp ΔMFE drop within the first 180 bp of the *b3952* N-terminal coding sequence under 15 distinct regulatory sequences.

Supplementary Table 1. Strains and plasmids used in this study

Strain or plasmid	Description	Reference or source
**Strain**		
E. coli DH5α	General cloning host	Lab stock
E. coli DB3.1	Cloning host for plasmids carrying the *ccdB* gene	Lab stock
E. coli MG1655	Wild-type strain	Lab stock
**Plasmid**		
pSC101-*ccdB*-GFP	pSC101-derived GFP expression plasmid, the regulatory sequence is replaced with a *Bsa*I-*ccdB*-*Bsa*I cassette to facilitate easy assembly	[1]
pSC101-*ccdB*-GFP-mCherry-opt	pSC101-*ccdB*-GFP derivative, carrying a mCherry expression cassette driven by promoter pL_M1-37. Two bidirectional transcriptional terminators were placed upstream and downstream of the GFP expression cassette	This study
pSC101-RSX-*ccdB*-mCherry-opt	A set of backbone plasmids to facilitate replacement of *ccdB* with the target genes (RSX denotes RS1-RS15)	This study
pSC101-RSX-*gfp*-mCherry-opt	A set of *gfp* expression plasmids, generated by replacement of *ccdB* with *gfp* in pSC101-RSX-*ccdB*-mCherry-opt	This study
pSC101-RSX-*lacZ*-mCherry-opt	A set of *lacZ* expression plasmids, generated by replacement of *ccdB* with *lacZ* in pSC101-RSX-*ccdB*-mCherry-opt	This study
pSC101-RSX-*icd*-mCherry-opt	A set of *icd* expression plasmids, generated by replacement of *ccdB* with *icd* in pSC101-RSX-*ccdB*-mCherry-opt	This study
pSC101-RSX-*zwf*-mCherry-opt	A set of *zwf* expression plasmids, generated by replacement of *ccdB* with *zwf* in pSC101-RSX-*ccdB*-mCherry-opt	This study
pSC101-RSX-*bfp*-mCherry-opt	A set of *bfp* expression plasmids, generated by replacement of *ccdB* with *bfp* in pSC101-RSX-*ccdB*-mCherry-opt	This study
pSC101-RSX-*gdhA*-mCherry-opt	A set of *gdhA* expression plasmids, generated by replacement of *ccdB* with *gdhA* in pSC101-RSX-*ccdB*-mCherry-opt	This study
pSC101-RSX-*icd-P.p*-mCherry-opt	A set of *icd-P.p* expression plasmids, generated by replacement of *ccdB* with *icd-P.p* in pSC101-RSX-*ccdB*-mCherry-opt	This study

pSC101-mCherry-opt	pSC101-*ccdB*-GFP-mCherry-opt derivative, removing the *ccdB* gene and GFP expression cassette	This study

Supplementary Table 2. Sequences used for synonymous mutation experiments

Gene	Feature	The 180-bp N-terminal coding sequence (5'-3')*
lacZ	amino acid sequence	MTMITDSLAVVLQRRDWENPGVTQLNRLAAHPPFASW<br>RNSEEARTDRPSQQLRSLNGEWR
	original DNA sequence	ATGACCATGATTACGGATTCACTGGCCGTCGTTTTACA<br>ACGTCGTGACTGGGAAAACCCTGGCGTTACCCAACTT<br>AATCGCCTTGCAGCACATCCCCCTTTCGCCAGCTGGCG<br>TAATAGCGAAGAGGCCCGCACCGATCGCCCTTCCCAA<br>CAGTTGCGCAGCCTGAATGGCGAATGGCGC
	mutant DNA sequence	ATGACGATGATCACAGACAGTTTAGCAGTTGTACTCC<br>AACGCCGCGACTGGGAGAATCCCGGTGTAACTCAATT<br>AAACCGTTTGGCCGCCCACCCTCCCTTTGCTTCATGGC<br>GCAACAGTGAGGAGGCACGTACCGACCGCCCAAGTCA<br>ACAACTTCGTTCTTTAAATGGTGAATGGCGC
icd	amino acid sequence	MESKVVVPAQGKKITLQNGKLNVPENPIIPYIEGDGIGVD<br>VTPAMLKVVDAAVEKAYKGE
	original DNA sequence	ATGGAAAGTAAAGTAGTTGTTCCGGCACAAGGCAAGA<br>AGATCACCCTGCAAAACGGCAAACTCAACGTTCCTGA<br>AAATCCGATTATCCCTTACATTGAAGGTGATGGAATC<br>GGTGTAGATGTAACCCCAGCCATGCTGAAAGTGGTCG<br>ACGCTGCAGTCGAGAAAGCCTATAAAGGCGAG
	mutant DNA sequence	ATGGAGTCAAAGGTCGTAGTACCTGCCCAAGGTAAGA<br>AGATTACTTTACAAAATGGTAAGTTGAATGTACCAGA<br>GAACCCTATCATTCCCTATATCGAGGGAGACGGGATT<br>GGAGTCGACGTCACCCCCGCCATGTTAAAGGTAGTAG<br>ACGCTGCCGTTGAGAAGGCGTACAAGGGTGAG
zwf	amino acid sequence	MAVTQTAQACDLVIFGAKGDLARRKLLPSLYQLEKAGQ<br>LNPDTRIIGVGRADWDKAAYTK
	original DNA sequence	ATGGCGGTAACGCAAACAGCCCAGGCCTGTGACCTGG<br>TCATTTTCGGCGCGAAAGGCGACCTTGCGCGTCGTAA<br>ATTGCTGCCTTCCCTGTATCAACTGGAAAAAGCCGGTC<br>AGCTCAACCCGGACACCCGGATTATCGGCGTAGGGCG<br>TGCTGACTGGGATAAAGCGGCATATACCAAA
	mutant DNA sequence	ATGGCAGTTACACAAACTGCTCAAGCGTGCGACTTAG<br>TTATCTTTGGTGCAAAGGGTGACCTTGCACGCCGCAA<br>GTTATTACCATCTTTATACCAATTAGAGAAGGCGGGA<br>CAACTTAATCCTGACACGCGGATTATTGGTGTAGGGC<br>GCGCTGACTGGGACAAGGCGGCCTACACCAAG
bfp	amino acid sequence	MVSKGEELIKENMHMKLYMEGTVDNHHFKCTSEGEGK<br>PYEGTQTMRIKVVEGGPLPFAFD

	original DNA sequence	ATGGTATCAAAAGGAGAAGAGCTAATAAAGGAGAAC<br>ATGCATATGAAATTGTACATGGAAGGCACCGTGGACA<br>ACCACCACTTCAAGTGCACCTCCGAAGGTGAAGGCAA<br>ACCGTATGAGGGCACCCAAACCATGCGTATTAAGGTG<br>GTCGAGGGCGGTCCGCTGCCGTTTGCGTTTGAC
	mutant DNA sequence	ATGGTCTCGAAGGGGGAGGAGCTAATAAAGGAGAAT<br>ATGCACATGAAGCTCTATATGGAGGGTACGGTCGACA<br>ATCATCATTTTAAGTGTACTTCGGAGGGGGAGGGTAA<br>GCCTTATGAGGGTACTCAAACTATGCGTATCAAGGTC<br>GTTGAGGGCGGCCCTCTGCCTTTTGCATTTGAC

* Mutated nucleotides in the mutant sequences are underlined.

Supplementary Table 3. Primers used in this study

**Plasmid or RT-PCR**		**Primer-F (5'-3')**	**Primer-R (5'-3')**
**pSC101-*ccdB*-GFP**	Fragment 1	CGGGCGGAGGTGGGTCGGGTGGCGGCGGATCAATGGGATCCATGTCGAAGGG	ATTTTAACTTGCTATTTCTAGCTCTAAAACCTACTTGTACAGCTCGTCC
	Fragment 2	GGACGAGCTGTACAAGTAGG	GGCGCCACGTCAGGTGGCAC
	Fragment 3	AAAAGTGCCACCTGACGTGG	ACCCGACCCACCTCCGCCCGAGCCTCCGCCACCTGAGACCTTATATTCCCCAG
**pSC101-*ccdB*-GFP-mCherry-opt**	Fragment 1	CTCTCCTGAGTAGGACAAATGCCTTTTTACGGTTCCTGGC	ATCGCCCATAGATCCTCCGCCACCTGAGACCTTATATTCCCCAG
	Fragment 2	GCGGAGGATCTATGGGCGATGGATCCATGTCGAAGGGCGA	TAGAGGTTCGTCGACGCGATCTACTTGTACAGCTCGTCCA
	Fragment 3	ATCGCGTCGACGAACCTCTAAGATCCGGCTGCTAACAAAG	TGGCAGCAGCCAACTCAGCT
	Fragment 4	AGCTGAGTTGGCTGCTGCCATTAGCATCCAAACTCGAGTAAGGATCT	GACAGTTTTCCCTTTGATATGTAACGGT
	Fragment 5	ATATCAAAGGGAAAACTGTCTTATCTCTGGCGGTGTTGACAAGA	ATTTGTCCTACTCAGGAGAGCGT
**pSC101-RS1-*ccdB*-mCherry-opt**	Fragment 1	ATCGCGTCGACGAACCTCTA	TGAGACCACGCGTGGATCCG
	Fragment 2	GGACCAAAACGAAAAAAGGC	TAGAGGTTCGTCGACGCGATTGAGACCTTATATTCCCCAG
	Fragment 3	GCCTTTTTTCGTTTTGGTCC	CGGATCCACGCGTGGTCTCATATGTATATCTCCTTCTTAAAAGATCTT
**pSC101-RS2-*ccdB*-mCherry-opt**	Fragment 1	ATCGCGTCGACGAACCTCTA	TGAGACCACGCGTGGATCCG
	Fragment 2	GGACCAAAACGAAAAAAGGC	TAGAGGTTCGTCGACGCGATTGAGACCTTATATTCCCCAG
	Fragment 3	GCCTTTTTTCGTTTTGGTCC	CGGATCCACGCGTGGTCTCATATGAAAGTCTCCTCCGCTA
**pSC101-RS3-*ccdB*-mCherry-opt**	Fragment 1	ATCGCGTCGACGAACCTCTA	TGAGACCACGCGTGGATCCG
	Fragment 2	GGACCAAAACGAAAAAAGGC	TAGAGGTTCGTCGACGCGATTGAGACCTTATATTCCCCAG

	Fragment 3	GCCTTTTTTCGTTTTGGTCC	CGGATCCACGCGTGGTCTCATATGGTTTTTCCACTCTTTCTC
**pSC101-RS4-*ccdB*-mCherry-opt**	Fragment 1	ATCGCGTCGACGAACCTCTA	TGAGACCACGCGTGGATCCG
	Fragment 2	GGACCAAAACGAAAAAAGGC	TAGAGGTTCGTCGACGCGATTGAGACCTTATATTCCCCAG
	Fragment 3	GCCTTTTTTCGTTTTGGTCC	CGGATCCACGCGTGGTCTCATATGAAACACTCCCTAGCTA
**pSC101-RS5-*ccdB*-mCherry-opt**	Fragment 1	ATCGCGTCGACGAACCTCTA	TGAGACCACGCGTGGATCCG
	Fragment 2	GGACCAAAACGAAAAAAGGC	TAGAGGTTCGTCGACGCGATTGAGACCTTATATTCCCCAG
	Fragment 3	GCCTTTTTTCGTTTTGGTCC	CGGATCCACGCGTGGTCTCATATGAAAAAATCCCCCGCTA
**pSC101-RS6-*ccdB*-mCherry-opt**	Fragment 1	ATCGCGTCGACGAACCTCTA	TGAGACCACGCGTGGATCCG
	Fragment 2	GGACCAAAACGAAAAAAGGC	TAGAGGTTCGTCGACGCGATTGAGACCTTATATTCCCCAG
	Fragment 3	GCCTTTTTTCGTTTTGGTCC	CGGATCCACGCGTGGTCTCATATGAAACGTTCCCTAGCTA
**pSC101-RS7-*ccdB*-mCherry-opt**	Fragment 1	ATCGCGTCGACGAACCTCTA	TGAGACCACGCGTGGATCCG
	Fragment 2	GGACCAAAACGAAAAAAGGC	TAGAGGTTCGTCGACGCGATTGAGACCTTATATTCCCCAG
	Fragment 3	GCCTTTTTTCGTTTTGGTCC	CGGATCCACGCGTGGTCTCATATGAAACTTTCCTCAGCTAG
**pSC101-RS8-*ccdB*-mCherry-opt**	Fragment 1	ATCGCGTCGACGAACCTCTA	TGAGACCACGCGTGGATCCG
	Fragment 2	GGACCAAAACGAAAAAAGGC	TAGAGGTTCGTCGACGCGATTGAGACCTTATATTCCCCAG
	Fragment 3	GCCTTTTTTCGTTTTGGTCC	CGGATCCACGCGTGGTCTCATATGAAACTCTCCCACGCTA
**pSC101-RS9-*ccdB*-mCherry-opt**	Fragment 1	ATCGCGTCGACGAACCTCTA	TGAGACCACGCGTGGATCCG

	Fragment 2	GGACCAAAACGAAAAAAGGC	TAGAGGTTCGTCGACGCGATTGAGACCTTATATTCCCCAG
	Fragment 3	GCCTTTTTTCGTTTTGGTCC	CGGATCCACGCGTGGTCTCATATGAAAATATCCCTAGCTAGATTA
**pSC101-RS10-*ccdB*-mCherry-opt**	Fragment 1	ATCGCGTCGACGAACCTCTA	TGAGACCACGCGTGGATCCG
	Fragment 2	GGACCAAAACGAAAAAAGGC	TAGAGGTTCGTCGACGCGATTGAGACCTTATATTCCCCAG
	Fragment 3	GCCTTTTTTCGTTTTGGTCC	CGGATCCACGCGTGGTCTCATATGGTAGTTCCACTCTTTCTC
**pSC101-RS11-*ccdB*-mCherry-opt**	Fragment 1	ATCGCGTCGACGAACCTCTA	TGAGACCACGCGTGGATCCG
	Fragment 2	GGACCAAAACGAAAAAAGGC	TAGAGGTTCGTCGACGCGATTGAGACCTTATATTCCCCAG
	Fragment 3	GCCTTTTTTCGTTTTGGTCC	CGGATCCACGCGTGGTCTCATATGGGCCTTTCCTGTGTGA
**pSC101-RS12-*ccdB*-mCherry-opt**	Fragment 1	ATCGCGTCGACGAACCTCTA	TGAGACCACGCGTGGATCCG
	Fragment 2	GGACCAAAACGAAAAAAGGC	TAGAGGTTCGTCGACGCGATTGAGACCTTATATTCCCCAG
	Fragment 3	GCCTTTTTTCGTTTTGGTCC	CGGATCCACGCGTGGTCTCATATGGTACGTCCAATCTTTC
**pSC101-RS13-*ccdB*-mCherry-opt**	Fragment 1	ATCGCGTCGACGAACCTCTA	TGAGACCACGCGTGGATCCG
	Fragment 2	GGACCAAAACGAAAAAAGGC	TAGAGGTTCGTCGACGCGATTGAGACCTTATATTCCCCAG
	Fragment 3	GCCTTTTTTCGTTTTGGTCC	CGGATCCACGCGTGGTCTCATATGAAAATATCCCTAGCTAGATTAA
**pSC101-RS14-*ccdB*-mCherry-opt**	Fragment 1	ATCGCGTCGACGAACCTCTA	TGAGACCACGCGTGGATCCG
	Fragment 2	GGACCAAAACGAAAAAAGGC	TAGAGGTTCGTCGACGCGATTGAGACCTTATATTCCCCAG
	Fragment 3	GCCTTTTTTCGTTTTGGTCC	CGGATCCACGCGTGGTCTCATATGAAAAAATCCCCCGCTA

**pSC101-RS15-*ccdB*-mCherry-opt**	Fragment 1	ATCGCGTCGACGAACCTCTA	TGAGACCACGCGTGGATCCG
	Fragment 2	GGACCAAAACGAAAAAAGGC	TAGAGGTTCGTCGACGCGATTGAGACCTTATATTCCCCAG
	Fragment 3	GCCTTTTTTCGTTTTGGTCC	CGGATCCACGCGTGGTCTCATATGAAAGTCTCCTCCGCTA
**pSC101-RSX-*gfp*-mCherry-opt**	*gfp* gene	ATGGGATCCATGTCGAAGGG	CTACTTGTACAGCTCGTCCAT
	backbone	ATCGCGTCGACGAACCTCTA	GGACCAAAACGAAAAAAGGC
**pSC101-RSX-*lacZ*-mCherry-opt**	*lacZ* gene	ATGACCATGATTACGGATTCACTGG	TTATTTTTGACACCAGACCAACTGGT
	backbone	ATCGCGTCGACGAACCTCTA	GGACCAAAACGAAAAAAGGC
**pSC101-RSX-*icd*-mCherry-opt**	*icd* gene	ATGGAAAGTAAAGTAGTTGTTCCGGC	TTACATGTTTTCGATGATCGCGTCAC
	backbone	ATCGCGTCGACGAACCTCTA	GGACCAAAACGAAAAAAGGC
**pSC101-RSX-*zwf*-mCherry-opt**	*zwf* gene	ATGGCGGTAACGCAAACAGC	TTACTCAAACTCATTCCAGGAACGAC
	backbone	ATCGCGTCGACGAACCTCTA	GGACCAAAACGAAAAAAGGC
**pSC101-RSX-*bfp*-mCherry-opt**	*bfp* gene	ATGGTATCAAAAGGAGAAGAGCTAATAAAGGAG	TTAGTTCAATTTGTGTCCAAGCTTGCTC
	backbone	ATCGCGTCGACGAACCTCTA	GGACCAAAACGAAAAAAGGC
**pSC101-RSX-*gdhA*-mCherry-opt**	*gdhA* gene	ATGGATCAGACATATTCTCTGGAGTCATTC	TTAAATCACACCCTGCGCCA
	backbone	ATCGCGTCGACGAACCTCTA	GGACCAAAACGAAAAAAGGC
**pSC101-RSX-*icd-P.p*-mCherry-opt**	*icd-P.p* gene	ATGGGATACCAGAAAATCAAGGTTCC	TTACATGTGTTTGATCATTTCATCGCCA
	backbone	ATCGCGTCGACGAACCTCTA	GGACCAAAACGAAAAAAGGC
**pSC101-mCherry-opt**	Fragment 1	TAGAGGTTCGTCGACGCGATGGACCAAAACGAAAAAAGGC	GGCCTACGTGAAGCACCCTG
	Fragment 2	ATCGCGTCGACGAACCTCTA	CAGGGTGCTTCACGTAGGCC
**RT-PCR**	GFP Fragment	TGGTCCTGCTGGAGTTCGTC	CAACTCAGCTTCCTTTCGGGC
	mCherry Fragment	GGGCGAGATCAAGCAGAGAC	CCAGCTTGATGTTCACGTTGT

REFERENCES

1 Wen, X. et al. Automated characterization and analysis of expression compatibility between regulatory sequences and metabolic genes in *Escherichia coli*. *Synth. Syst. Biotechnol*. **9**, 647-657 (2024).
